# Functional and Pangenomic Exploration of Roc Two‐Component Regulatory Systems Identifies Novel Players Across *Pseudomonas* Species

**DOI:** 10.1111/mmi.15357

**Published:** 2025-03-14

**Authors:** Victor Simon, Julian Trouillon, Ina Attrée, Sylvie Elsen

**Affiliations:** ^1^ Institute of Structural Biology, UMR5075, Team Bacterial Pathogenesis and Cellular Responses University Grenoble Alpes Grenoble France; ^2^ Institute of Molecular Systems Biology, ETH Zürich Zürich Switzerland

**Keywords:** comparative genomics, cup fimbriae, regulatory network, roc, two‐component system

## Abstract

The opportunistic pathogen *Pseudomonas aeruginosa* relies on a large collection of two‐component regulatory systems (TCSs) to sense and adapt to changing environments. Among them, the Roc (regulation of *cup*) system is a one‐of‐a‐kind network of branched TCSs, composed of two histidine kinases (HKs—RocS1 and RocS2) interacting with three response regulators (RRs—RocA1, RocR, and RocA2), which regulate virulence, antibiotic resistance, and biofilm formation. Based on extensive work on the Roc system, previous data suggested the existence of other key regulators yet to be discovered. In this work, we identified PA4080, renamed RocA3, as a fourth RR that is activated by RocS1 and RocS2 and that positively controls the expression of the *cupB* operon. Comparative genomic analysis of the locus identified a gene—*rocR3*—adjacent to *rocA3* in a subpopulation of strains that encodes a protein with structural and functional similarity to the c‐di‐GMP phosphodiesterase RocR. Furthermore, we identified a fourth branch of the Roc system consisting of the PA2583 HK, renamed RocS4, and the Hpt protein HptA. Using a bacterial two‐hybrid system, we showed that RocS4 interacts with HptA, which in turn interacts with RocA1, RocA2, and RocR3. Finally, we mapped the pangenomic RRs repertoire, establishing a comprehensive view of the plasticity of such regulators among clades of the species. Overall, our work provides a comprehensive inter‐species definition of the Roc system, nearly doubling the number of proteins known to be involved in this interconnected network of TCSs controlling pathogenicity in *Pseudomonas* species.

## Graphical abstract

The Roc system represents a highly interconnected but incomplete network of two‐component regulatory systems involved in the virulence of *Pseudomonas aeruginosa* . Our work has enabled us to identify the missing RocA3 regulator, to propose new players in the system and to delineate their conservation between clades of the species.

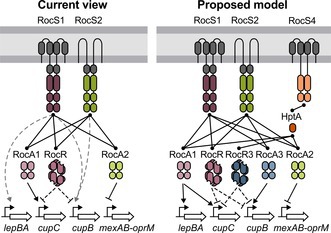

## 1 Introduction

*Pseudomonas aeruginosa* is an opportunistic pathogen responsible for a wide range of human infections, whose variability in virulence and antibiotic resistance capacity relies mainly on the high heterogeneity of its large genomes (Klockgether and Tümmler 2017). Phylogenetic analyses determined the population structure of the species, identifying five different clades with, in particular, distinct pathogenesis strategies (Freschi et al. 2019; Ozer et al. 2019; Quiroz‐Morales et al. 2022). Clade 3 includes the PA7‐like strains known as taxonomic outliers (Roy et al. 2010), which have been recently reclassified as a new species, *P. paraeruginosa* (Rudra et al. 2022). The ability of these species to cause disease and survive in diverse environments is largely due to sophisticated transcriptional regulatory networks, of which two‐component regulatory systems (TCSs) are key players (Galán‐Vásquez et al. 2011; Francis et al. 2017).

TCSs are widespread signaling machineries that allow bacteria to directly respond to environmental cues and adapt to diverse conditions (Stock et al. 2000; Capra and Laub 2012; Zschiedrich et al. 2016). As their name suggests, classical TCSs are a pair of signal transduction proteins communicating by phosphotransfer: a sensor histidine kinase (HK), commonly transmembrane, and a cytoplasmic response regulator (RR). HKs are often bifunctional enzymes with phosphatase and autokinase activities that are modulated by one or more chemical or physical signals, most of which are still unknown (Zschiedrich et al. 2016). The HK typically autophosphorylates a conserved histidine residue within its transmitter domain (H domain), and the phosphoryl group is then transferred to a conserved aspartate residue on the receiver domain (D or REC domain) of the RR, resulting in its activation (Stock et al. 2000). Since most RRs are transcription factors, the output response is often the modulation of target gene expression.

In addition to the typical TCSs with a two‐step phosphoryl transfer, there are many reports of four‐step phosphorelays where the phosphoryl group is transferred between additional domains on the HK or other protein partners (Stock et al. 2000; Zschiedrich et al. 2016). For instance, an “unorthodox” HK has two additional C‐terminal domains, a receiver domain and a histidine‐containing phosphotransfer (Hpt) domain in a so‐called H1–D1–H2 organization. In this case, internal phosphoryl transfer along the H1–D1–H2 domains precedes phosphorylation of the corresponding RR, as exemplified by GacS in *P. aeruginosa* , which plays a crucial role in the transition from chronic to acute infection (Goodman et al. 2004). Four‐step phosphorelays also occur in the case of a “hybrid” HK that contains an additional D1 receiver domain and requires an external Hpt protein to phosphorylate the RR. In some cases, multiple interacting partners are involved, where a RR can be activated by several HKs or where one HK can activate several RRs, further increasing the complexity of these systems (Francis and Porter 2019). Gene duplication, with subsequent divergence, and acquisition by horizontal gene transfer (HGT) are the main sources of new pathways (Alm et al. 2006; Capra and Laub 2012). *In silico* modeling suggests that rapid isolation of signaling between newly duplicated TCSs is necessary to limit non‐cognate HK and RR interactions (i.e., cross‐talk) that are a potential burden on cell fitness (Capra and Laub 2012; Rowland and Deeds 2014).

Approximately 130 genes that code for TCS partners have been predicted in the genome of *P. aeruginosa* strain PAO1 (Rodrigue et al. 2000), which emerged through both recruitment and gene duplication events (Chen et al. 2004). Many of these TCSs have been shown to be involved in key infection‐related processes, including motility, biofilm formation, cytotoxicity, virulence, and antibiotic resistance (Francis et al. 2017; Sultan et al. 2021). Among them, the Roc (regulation of *cup*) signaling pathway in *P. aeruginosa* stands out for its large number of interconnected players. It consists of two paralogous unorthodox HKs (RocS1 and RocS2) interacting with three RRs (RocA1, RocA2 and RocR) (Kulasekara et al. 2004; Kuchma et al. 2005; Sivaneson et al. 2011). The proteins are encoded by two distinct genetic loci, the Roc1 system by *rocA1* and the divergently transcribed *rocR‐rocS1* operon, and the Roc2 system by the *rocA2‐rocS2* operon. Despite these differences in genetic organization, phylogenetic studies indicated that the two loci evolved by gene duplication and probable gene rearrangement (Chen et al. 2004). This common origin may underlie why both HKs RocS1 and RocS2 can interact with and activate all three Roc RRs (Kulasekara et al. 2004; Sivaneson et al. 2011). Of these three RRs, RocA1 and RocA2 are transcription factors, and RocR is a c‐di‐GMP phosphodiesterase (Kulasekara et al. 2004; Rao et al. 2008; Sivaneson et al. 2011). The main targets of the Roc system are two operons encoding Cup (chaperone‐usher pathway) fimbriae, surface appendages that participate in biofilm formation through adhesion to abiotic surfaces, cell–cell interaction, and microcolony formation (Ruer et al. 2007; Giraud et al. 2009, 2011). Both the *cupC* and *cupB* operons were shown to be activated in a RocS1‐ and RocS2‐dependent manner. However, whereas RocA1 controls *cupC* expression, none of the three known Roc RRs account for *cupB* activation; this suggests the existence of an unknown additional regulator in the Roc system (Kulasekara et al. 2004; Sivaneson et al. 2011).

Recently, we explored the global TCSs regulatory network in different *Pseudomonas* strains by characterizing in vitro DNA‐binding sites for 55 RRs using DAP‐seq (DNA affinity purification and sequencing) (Trouillon et al. 2021). The study was carried out on three reference strains, *P. aeruginosa* PAO1 and PA14 (Freschi et al. 2019), and *P. paraeruginosa* IHMA87 (Kos et al. 2015), allowing the intra‐ and interspecies comparison of regulatory networks through the 48 regulators that are conserved within the three strains (Trouillon et al. 2021). In the present study, we reexamined the DAP‐seq data to find the RRs that bind to the promoter of the *cupB* operon and found that the uncharacterized orphan RR PA4080 exhibited one of the strongest bindings on the PA14 genome (Trouillon et al. 2021). We experimentally demonstrated that PA4080, whose gene is located downstream of the *cupB* operon, activates this operon in a RocS1‐ and RocS2‐dependent manner, and was therefore named RocA3. Using comparative genomics, we identified several new putative partners, either conserved or restricted to a few clades of *P. aeruginosa* and *P. paraeruginosa*. Our work expands our understanding of the Roc system and highlights how such interconnected TCSs contribute to the diversity of regulatory networks in these species.

## 2 Results

### 2.1 PA4080 is an Activator of the *cupB* Operon

**FIGURE 1:**
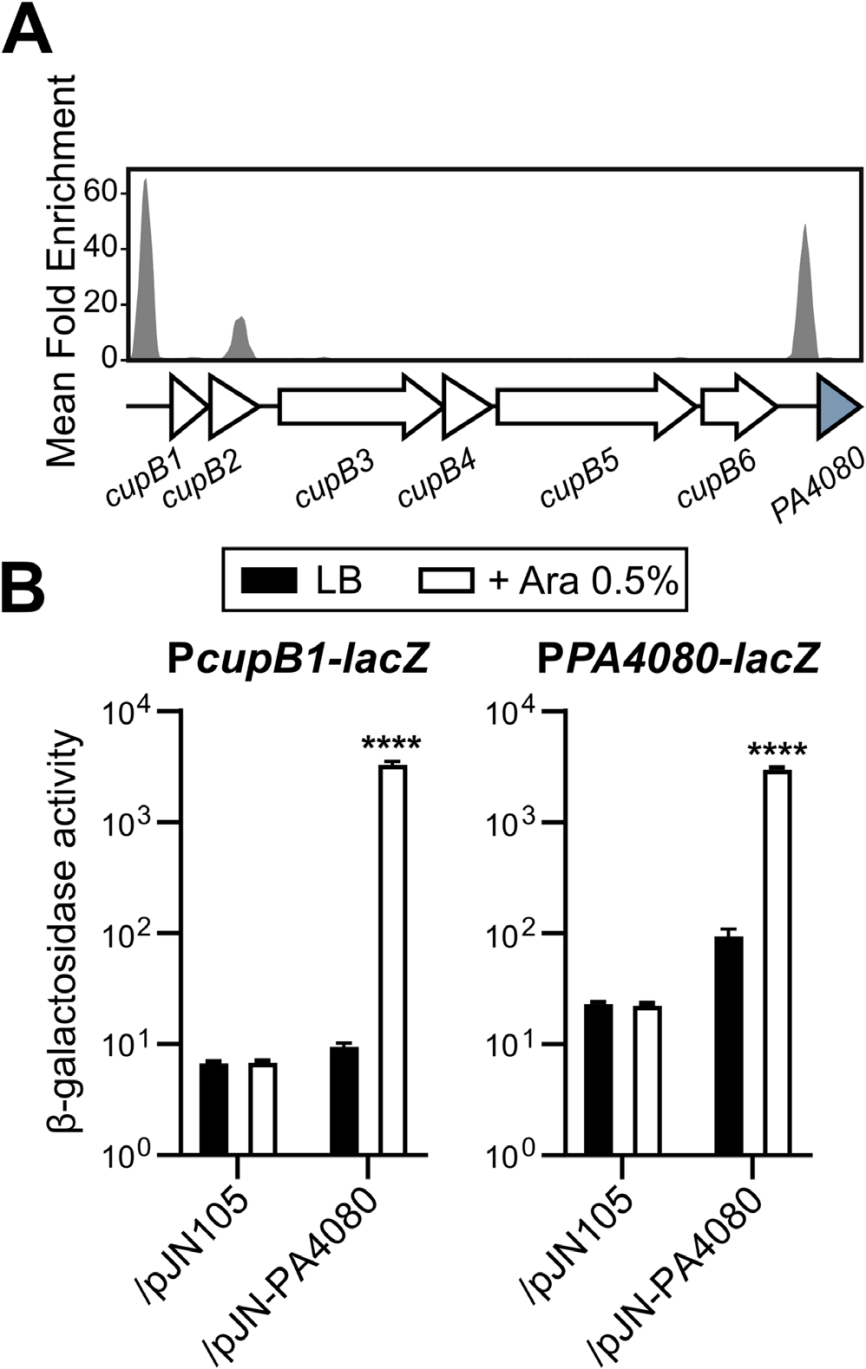
PA4080 regulates the *cupB* operon and its own expression (A) Reanalyses of previously published data on the PA14 genome (Trouillon et al. 2021) showing the enrichment coverage track of DAP‐seq against the negative control for the RR PA4080 (PA14_11120) at the indicated locus. (B) β‐galactosidase activities of the indicated strains harboring the P*cupB1‐lacZ* and P*PA4080‐lacZ* transcriptional fusions. The strains also carried either the empty pJN105 or the pJN‐PA4080 plasmid, and expression of *PA4080* was induced with 0.5% arabinose for 2.5 h in LB medium. Experiments were performed in triplicate and the error bars represent the SEM. Statistical analysis was performed using two‐way ANOVA, followed by Dunnett's test for comparison with the control condition (PAO1 WT/pJN105 in LB). ****p < 0.0001.

Previous DAP‐seq data (Trouillon et al. 2021) were re‐analysed to find potential RRs that control the expression of the *cupB* operon. One of the strongest peak enrichments upstream of the *cupB1* gene, the first gene of the *cupB* operon, was observed for RR PA4080 (Figure 1A), with the binding signal centered at 214 bp from the coding sequence (Figure S1A) (Trouillon et al. 2021). This binding was only observed on the PA14 genome as DAP‐seq with PA4080 did not provide enriched targets on the PAO1 and IHMA87 genomes, which may be due to a technical problem with the samples. The *PA4080* gene, which is conserved across *P. aeruginosa* and *P. paraeruginosa* strains, is encoded downstream of the *cupB* operon, and this physical proximity strengthens a hypothetical regulatory link (Lawrence 2003). To first assess the regulatory role of PA4080 on the expression of the *cupB* operon, a transcriptional *lacZ* fusion with the *cupB1* promoter (P*cupB1*‐*lacZ*) was constructed and integrated into the PAO1 chromosome, while *PA4080* was overexpressed through the arabinose‐inducible P*BAD* promoter on the pJN105 replicative plasmid. As previously observed for the different *cup* operons (Kulasekara et al. 2004), the *cupB* operon was poorly expressed under laboratory conditions, with a level of β‐galactosidase activity of the transcriptional fusion below 10 Miller units (Figure 1B). However, overexpression of *PA4080* strongly induced *cupB1* expression, with a 350‐fold increase in reporter activity, demonstrating that PA4080 was an activator of this operon. Since the RR was also able to bind upstream of its own gene in vitro (Figure 1A), we used a similar approach and observed that PA4080 positively autoregulates its own gene (Figure 1B). Two known targets of the Roc system controlled by either RocA1 or RocA2 were also tested by generating and analyzing the expression of P*cupC1*‐*lacZ* (activated by RocA1; Kulasekara et al. 2004) and P*mexA*‐*lacZ* (inhibited by RocA2; Sivaneson et al. 2011). Our data showed that PA4080 does not control the expression of the *cupC* and *mexAB*‐*oprM* operons (Figure S1B).

Taken together, our results demonstrated that PA4080 is a direct activator of the *cupB* operon and its own gene. Considering the incomplete regulatory system already known to activate *cupB* expression, we hypothesized that PA4080 is the suspected additional regulator of the Roc system.

### 2.2 PA4080 Belongs to the Roc System

**FIGURE 2:**
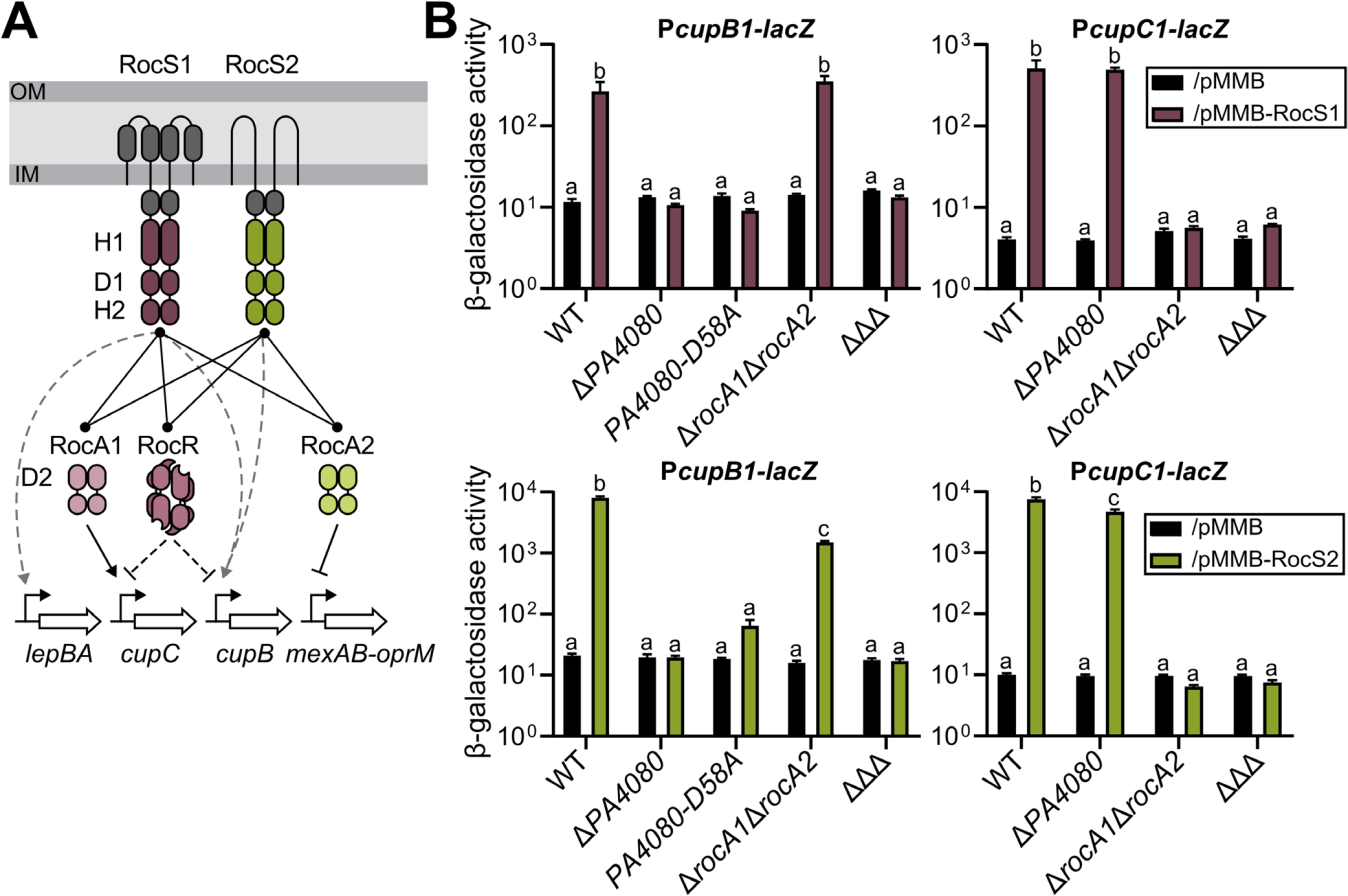
PA4080 is the missing RocA3 regulator (A) Current view of the Roc signaling pathways. RocR inhibits indirectly expression of *cupC* and *cupB* through c‐di‐GMPc degradation. Grey dashed lines represent activation through unidentified regulator. RocR is depicted a tetrameric structure as determined by Chen et al. (2012). Domains shown are according to PFAM nomenclature; SBP_bac_3 (periplasmic grey domain), PAS_4 (cytosolic grey domain), HisKA and HATPase_c (H1), Response_reg (D1 and D2), Hpt (H2) and either GerE (DNA‐binding domain: Plain shape) or EAL (c‐di‐GMP phosphodiesterase; hollow shape) for output domain of RRs. (B) β‐galactosidase activities of the strains and mutants carrying the P*cupB1‐lacZ* and P*cupC1‐lacZ*, as indicated. The strains also carried either the empty pMMB plasmid (black bars), pMMB‐RocS1 (burgundy bars) or pMMB‐RocS2 (green bars) and the expression of the HKs was induced with IPTG for 6 h in M63 medium. ΔΔΔ corresponds to the triple mutant Δ*rocA1*Δ*rocA2*Δ*PA4080*. The experiments were performed in triplicate and the error bars represent the SEM. Different letters indicate significant differences according to two‐way ANOVA followed by Tukey's multiple comparison test (*p* < 0.05).

To determine whether PA4080 belongs to the Roc system, we assessed its possible activation by the two HKs, RocS1 and RocS2 (Figure 2A). To do this, we artificially activated the Roc system by overproducing either RocS1 or RocS2, as previously described (Kulasekara et al. 2004; Sivaneson et al. 2011). As expected, using chromosomal transcriptional *lacZ* fusions, these overexpressions led to an increased activity of P*cupB1* and P*cupC1* compared to the strains carrying empty vectors in the wild‐type genetic background (Figure 2B). The fusions were then introduced into a *PA4080* gene deletion mutant, a double mutant Δ*rocA1*Δ*rocA2*, and a strain lacking the three RRs (Δ*rocA1*Δ*rocA2*Δ*PA4080*). Deletion of *PA4080* completely abolished the *cupB* operon induction triggered by either HK overproduction, indicating that *cupB* expression is entirely dependent on PA4080 activity and that this RR is the previously unidentified regulator responsible for Roc‐dependent regulation of *cupB* (Sivaneson et al. 2011). In the Δ*rocA1*Δ*rocA2* mutant, activation of *cupB1* was similar to that observed in the wild‐type when *rocS1* was overexpressed, in agreement with previous studies (Kulasekara et al. 2004; Sivaneson et al. 2011). However, a small but significant reduction in *cupB1* expression was observed upon RocS2 overproduction in the double mutant (Figure 2B), even though RocA1 and RocA2 are not direct regulators of the *cupB* operon. These effects, observed only with the HK inducing the highest activities, may result from interruption of autoregulatory loops, as observed for PA4080 (Figure 1B) and RocA1 (Kulasekara et al. 2004). Finally, as expected, a complete loss of *cupB* induction was observed in the absence of the three RRs.

We further examined the expression of the *cupC* operon under conditions of RocS1 or RocS2 overproduction in these different mutant backgrounds. Consistent with the absence of control of PA4080 on this promoter (Figure S1B), inactivation of *PA4080* did not affect the activation of *cupC1* expression when *rocS1* was overexpressed. However, we observed a small but significant reduction in *cupC1* expression upon RocS2 overproduction in the *PA4080* mutant, which again may be indirect. To confirm that the *cupB1* induction was due to the activation of PA4080 by HK phosphorylation, a mutation was introduced in *PA4080* to change the conserved aspartate residue of the regulator to an alanine residue (D58A), preventing RR phosphorylation (Figure 2B). This mutation drastically reduced the activation of *cupB1* expression when RocS1 and RocS2 were overproduced, indicating that this induction requires a phosphorylated PA4080 protein.

Overall, our results indicate that PA4080 is part of the Roc system that mediates the activation of the *cupB* operon by both RocS1 and RocS2 sensory kinases. We have therefore named PA4080 as RocA3.

### 2.3 RocA1 Controls the Expression of the *lepBA* Operon

**FIGURE 3:**
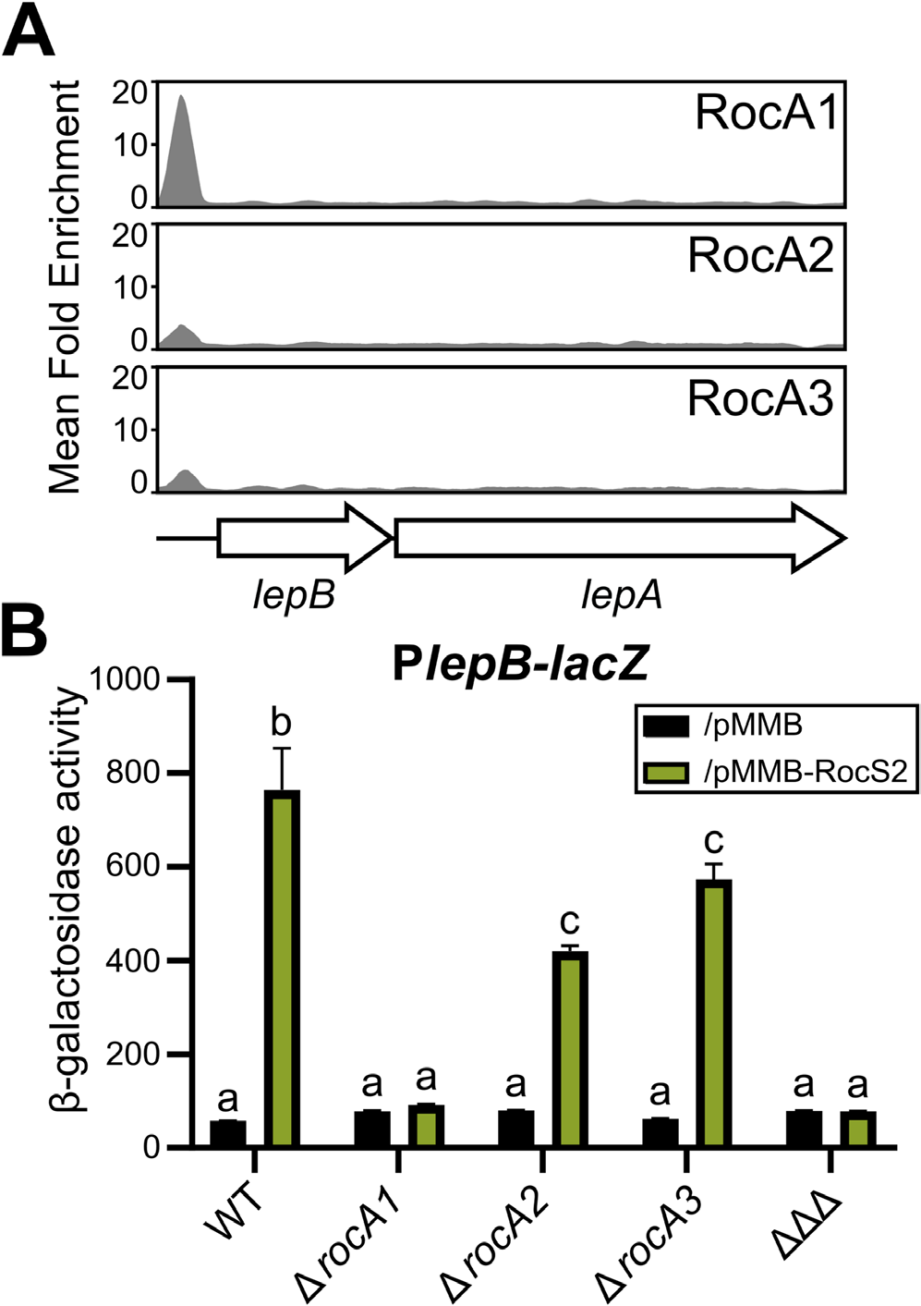
RocA1 controls the synthesis of the LepBA system (A) Re‐analysis of previously published data on the PA14 genome (Trouillon et al. 2021) showing the enrichment coverage track of DAP‐seq against the negative control for the three RocA regulators on the *lepBA* upstream region. (B) β‐galactosidase activities of the strains and mutants carrying the chromosome‐integrated P*lepB‐lacZ*. Strains also carried either the empty pMMB (black bars) or pMMB‐rocS2 (green bars) plasmid and HK expression was induced with IPTG for 6 h in M63 medium. ΔΔΔ corresponds to the triple mutant Δ*rocA1*Δ*rocA2*Δ*rocA3*. Experiments were performed in triplicate and the error bars represent the SEM. Different letters indicate significant differences according to two‐way ANOVA followed by Tukey's multiple comparison test (*p*‐value < 0.05).

In addition to controlling fimbriae and MexAB‐OprM synthesis, the Roc system was reported to regulate *lepBA*, as this operon is activated when RocS1 is overproduced. LepBA is a multi‐effector secretion system in which LepB transports both the LepA protease and CupB5, encoded in the *cupB* operon, across the outer membrane (Garnett et al. 2015). Given this functional link, we suspected that the newly identified RocA3 might be responsible for the activation of *lepBA* expression in addition to that of *cupB*. From the DAP‐seq data, we observed that the *lepB* promoter region was indeed targeted in vitro by RocA3, but also by the other two RocA proteins, with the higher peak observed for RocA1 (Figure 3A). We therefore investigated the potential regulatory contributions of the three RRs using a P*lepB*‐*lacZ* chromosomal transcriptional fusion during the activation of the Roc system by *rocS2* overexpression. We first observed a strong activation of *lepB* expression in the wild‐type genetic background when RocS2 was overproduced (Figure 3B). Deletion of *rocA1* almost completely abolished this up‐regulation, suggesting that RocA1 is the main Roc activator of the *lepBA* operon, consistent with the strong DNA binding observed for this RR to the promoter in vitro (Figure 3A). Although the effects were small, deletions of *rocA2* and *rocA3* also had a negative effect on the activation of *lepBA* expression by *rocS2* overexpression, which could be explained by their lower binding intensities observed in vitro (Figure 3A) or by small indirect effects on RocA1 synthesis or activation.

Re‐analysis of the DAP‐seq data (Trouillon et al. 2021) enabled us to define the consensus DNA‐binding sequences of the three RocA RRs using MEME‐ChIP and found them to be highly similar (Figure S2A). Consistent with this observation, we found a large overlap in their putative in vitro gene targets, a fraction of which were shared by the three RRs (Figure S2B,C); these included the *roc1* locus, the *cupB* and *cupC* operons, and the *lepBA* operon, although they were shown to be mostly dependent on a single RocA regulator in vivo (Sivaneson et al. 2011; Figures 2, 3). This suggests that RocA1, RocA2, and RocA3 have specific targets despite their similar DNA‐binding sequences. Identified in vitro DNA motifs are prone to inaccuracies due to false positives inherent in the method, and discriminating differences between the three RRs may have been overlooked. Of note, RocA2 is the response regulator with the fewest targets, and in the absence of *mexAB‐oprM* as a direct target, its mechanism of regulation of the antibiotic resistance phenotype remains unknown.

In conclusion, the Roc system unexpectedly relies on two RRs, RocA1 and RocA3, to orchestrate the expression of the functionally related proteins LepB and CupB5, respectively.

### 2.4 Organization and Diversity of the New *roc3* Locus

**FIGURE 4:**
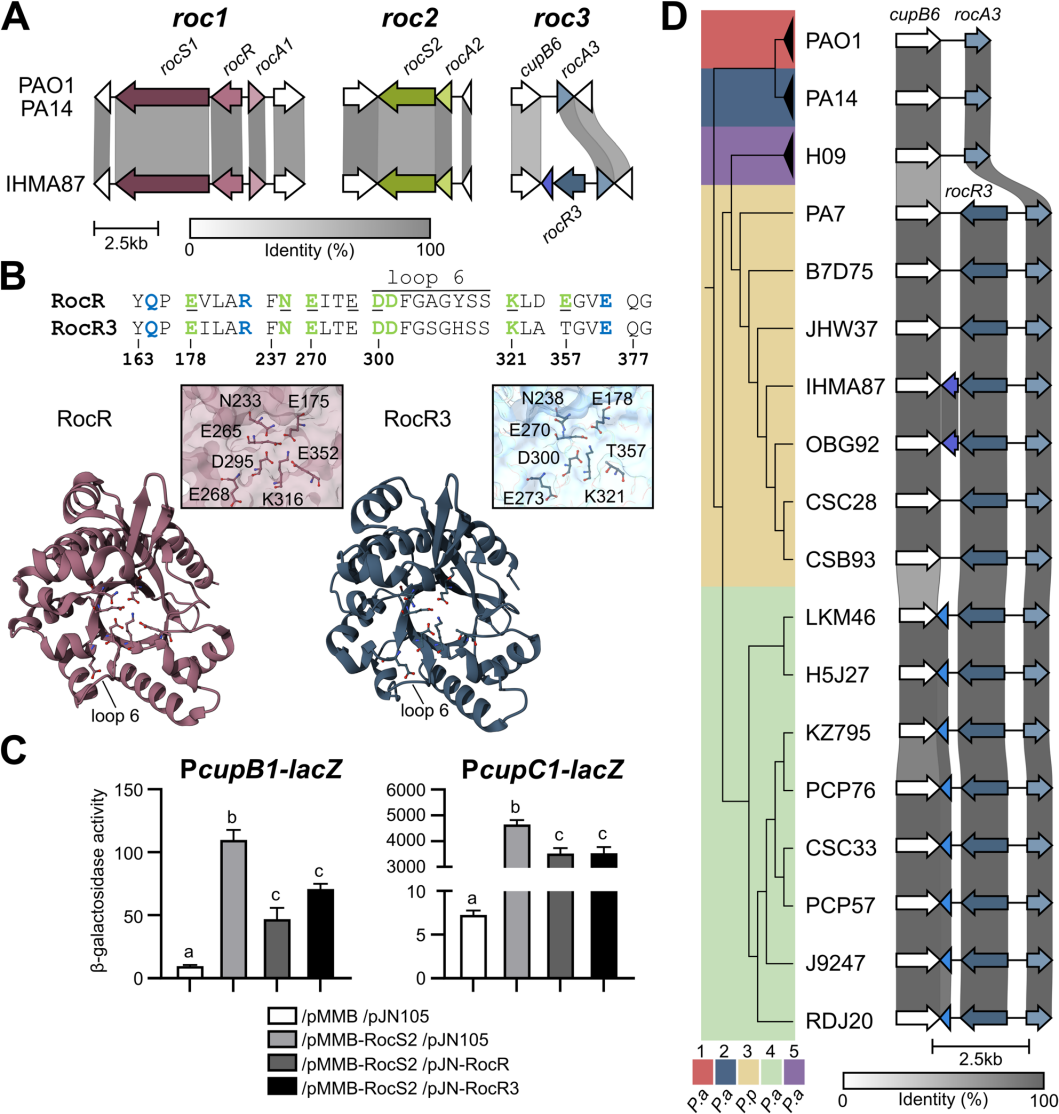
The *roc3* locus in *Pseudomonas aeruginosa and Pseudomonas paraeruginosa* species (A) Loci encoding the three Roc systems are represented in the *P. aeruginosa* PAO1 and PA14 strains and *P. paraeruginosa* IHMA87, with the percentage of sequence identity indicated by the grey scale. The genes located on either side of the *roc* genes are also indicated to show the conservation of their location. In the IHMA87 Roc3 system, *rocR3* is homologous to *rocR* while the downstream pseudogene may encode part of an H1 domain, which is an Hpt‐like module. (B) Comparison of RocR and RocR3. The upper section shows conserved motifs, with amino acids involved in substrate binding colored in blue and those involved in metal ion coordination colored in green. The seven amino acids identified as essential for catalysis (Rao et al. 2009) are underlined (adapted from Römling 2009). The lower section shows an upper view of the structure of the EAL domain of a RocR monomer (3SY8) compared to the model proposed by RoseTTAFold for the EAL domain of RocR3. Details of the organisation of the seven essential residues are shown alongside. The model was obtained using the https://robetta.bakerlab.org/submit.php server, accessed in May 2024 and visualised with Mol* 3D Viewer (Sehnal et al. 2021). (C) β‐galactosidase activities of the PAO1 strain containing the P*cupB‐lacZ* or P*cupC‐lacZ* transcriptional fusion. The strains carried two replicative plasmids as indicated below. Gene expression was induced with 1 mM IPTG and 0.2% arabinose for 6 h in M63 medium. Experiments were performed in triplicate and the error bars represent the SEM. Different letters indicate significant differences according to two‐way ANOVA followed by Tukey's multiple comparison test (*p* < 0.05). (D) Genetic comparison of *roc3* loci in *rocR3* ^+^ strains with the percentage of sequence identity indicated by the grey scale. PAO1, PA14, and H09 were used as reference for clades 1, 2, and 5, respectively. Sequences were ordered based on the genomic phylogeny shown.

In PAO1 and PA14, the *rocA3* gene is located adjacent to the *cupB6* gene in a region distinct from the *roc1* and *roc2* loci, which we named the *roc3* locus (Figure 4A). We noted that two additional genes are predicted in this locus, between *cupB6* and *rocA3*, in *P. paraeruginosa* IHMA87 (Winsor et al. 2016) (Figure 4A). One is a hypothetical coding sequence (CDS) (*IHMA87*_*RS04355*) which encodes a protein with a predicted Hpt domain, suggesting a potential role in a TCS phosphorelay. The second gene encodes a putative RR (IHMA87_00844, named RocR3 here) that, like RocR, possesses a C‐terminal EAL domain that is predicted to have a c‐di‐GMP‐specific phosphodiesterase activity. The *P. aeruginosa* RocR protein has been reported to be a negative regulator of the Roc1 and Roc2 subsystems, inhibiting expression of the *cupB* and *cupC* operons probably indirectly, through degradation of the c‐di‐GMP second messenger (Figure 2A) (Kulasekara et al. 2004; Rao et al. 2008; Sivaneson et al. 2011). The two proteins share 49% sequence identity, and a structural model of RocR3 revealed a similar architecture to that of RocR (Chen et al. 2012) (Figure 4B). However, one of the seven conserved residues identified as essential for the catalytic activity of the EAL domain in RocR was not conserved in RocR3 (E357T) (Rao et al. 2008) and the motif of loop 6, which is also involved in the activity (Rao et al. 2009), showed two differences (A304S and Y306H), suggesting that the phosphodiesterase activity of the protein might be compromised.

To assess the role of RocR3 in the Roc network, we compared the effect of RocR and RocR3 overproduction in strains harboring the P*cupB* and P*cupC* transcriptional *lacZ* fusions in the presence of pMMB‐RocS2. As expected, RocR overproduction significantly limited the expression of both operons (Figure 4C). Strikingly, the expression of both operons was also significantly reduced when RocR3 was overproduced, suggesting that the protein is also able to partially antagonize the activation of the RocA1‐controlled *cupC* and RocA3‐controlled *cupB* operons by a mechanism to be determined.

The IHMA87 *roc3* locus is reminiscent of the *roc1* locus in its organization, with two divergent genes encoding two RRs: the transcription factor RocA3 and the EAL domain protein RocR3, except for the absence of *rocS1* (Figure 4A). We examined the conservation of the *roc3* region in 804 complete genomes of *P. aeruginosa* (*n* = 793) and *P. paraeruginosa* (*n* = 7) available in the NCBI genome database. Each genome was grouped into a clade based on phylogeny, using previously established nomenclature (Freschi et al. 2019), with the outlier clade 3 now corresponding to *P. paraeruginosa* (Rudra et al. 2022) (Figure S3). First, we found that the synteny around *rocR3* was conserved in *P. aeruginosa* strains of clade 4 and in *P. paraeruginosa* strains (Figure 4D). Except for two clade 1 strains carrying a gene encoding a transposase, no other genes were predicted between *cupB6* and *rocA3* in the two main phylogenetic clades (1 and 2) and in clade 5 of *P. aeruginosa* . The adjacent Hpt‐encoding gene mentioned above was found in IHMA87 and OBG92 strains but not in other *P. paraeruginosa* strains, although the sequences are highly similar. The prediction of the gene in these two strains results from a 4 bp deletion in the CDS that shifts a stop codon in frame (Figure S4). All clade 4 strains carry a predicted gene next to *rocR3* encoding a HisKA domain that shows high sequence similarity to the H1 domain of RocS1 (Figure 4D). These results led us to the hypothesis that a gene encoding an unorthodox HK (like RocS1) was present downstream of *rocR3* but was lost during evolution. The deletion of this putative *rocS3* gene left different genetic scars between the clades, with residual information leading to the prediction of genes coding for parts of this ancestral HK (H1 domain in clade 4 and a Hpt domain reminiscent of an H2 domain in some clade 3 strains) (Figure S4).

In conclusion, an additional RR of the Roc system was identified in the *P. paraeruginosa* IHMA87 strain, RocR3, present in the newly named *roc3* locus, which harbors the *rocA3* core gene. The *roc3* locus presents clade‐specific gene organization, suggesting gene erosion during species evolution and divergence.

### 2.5 Synteny of the *cupC* Locus Suggests Additional Partners

**FIGURE 5:**
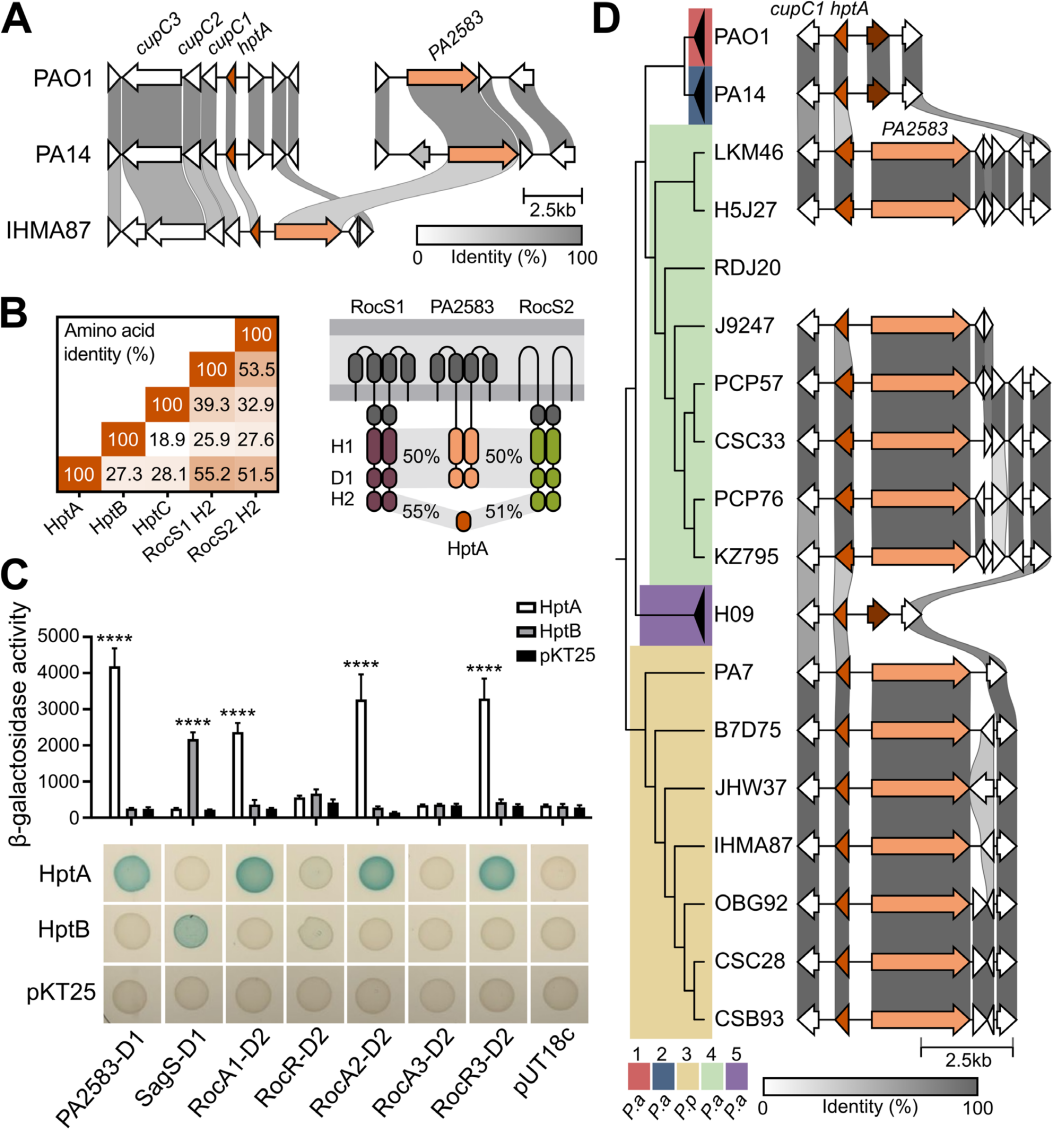
Additional putative players in the Roc system (A) Genetic organisation of the loci containing the *cupC* operon and the *PA2583* gene in the *P. aeruginosa* PAO1 and PA14 strains and *P. paraeruginosa* IHMA87, with the percentage of sequence identity indicated by the grey scale. (B) The percentage of amino acid identity of the three Hpt modules of PAO1 with the H2 domains of RocS1 and RocS2. The figure on the right shows the homologies of RocS1 and RocS2 unorthodox HKs, PA2583 hybrid HK and HptA proteins. H1, D1, and H2 correspond to the transmitter (HisKA/HATPAse_C), receiver and Hpt domains, respectively. The putative sensory domains are represented by periplasmic grey boxes for the Sbp3 domains and a cytoplasmic grey box for the PAS domain. (C) Bacterial two‐hybrid analysis of different combinations of recombinant pKT25 and pUT18c plasmids encoding full or partial proteins of interest in *E. coli* DHM1 spotted on LB agar containing X‐Gal and IPTG. Blue colored colonies indicate interacting proteins. β‐galactosidase activities of the resuspended spots from bacterial two‐hybrid analysis plates are shown on the right. Experiments were performed in triplicate and the error bars represent the SEM. Statistical analysis was performed using two‐way ANOVA, followed by Dunnett's test for comparison with the control condition (pKT25). ****p < 0.0001. (D) Genetic comparison of the *hptA* locus in strains harbouring PA2583 in the vicinity, with the percentage of sequence identity indicated by the grey scale. PAO1, PA14 and H09 were used as reference for clades 1, 2, and 5, respectively. Sequences were ordered based on the genomic phylogeny shown.

In the PAO1 and PA14 genomes, upstream of the Roc‐regulated *cupC* operon lies a gene encoding one of the three Hpt proteins, HptA (Figure 5A) (Winsor et al. 2016). As mentioned above, these Hpt proteins are intermediates for the phosphotransfer between hybrid HKs and their cognate RRs. However, neither of these proteins were encoded in the vicinity of the *hptA* gene in these two genomes. To assess whether a potential partner of HptA is present in other strains, we used comparative genomics to analyze the genetic environment around this gene in the different *P. aeruginosa* and *P. paraeruginosa* species. In the *P. paraeruginosa* strain IHMA87, we found that a gene (*IHMA87_RS21720*) encoding an orphan hybrid HK is located upstream of the *hptA* gene. The orthologous gene in the genomes of PAO1 (*PA2583*) and PA14 (*PA14*_*30700*) is located elsewhere between two tRNA genes, suggesting that these regions have undergone gene rearrangement (Figure 5A). Like RocS1, PA2583 possesses two predicted periplasmic sensor domains found in solute‐binding proteins (SBP_bac_3), which may be involved in ligand binding, and a cytoplasmic PAS domain (Kulasekara et al. 2004). Although PA2583 is a hybrid HK and thus differs from RocS1 in the absence of an additional H2 domain, they both share high sequence identities within their H1 and D1 domains (Figure 5B). In addition, their phylogenetic proximity was previously revealed by Chen et al. (2004), with PA2583 being the closest HK to RocS1 and RocS2, as mentioned by Sivaneson et al. (2011). As the *hptA* gene is contiguous to the gene encoding the predicted hybrid HK PA2583 in IHMA87, we hypothesized that they could be part of a phosphorelay integrated into Roc TCSs. This was supported by the observation that the H2 domain of HptA is very similar to those of RocS1 and RocS2, sharing more than 50% amino acid sequence identity with them. In comparison, the sequence identity of HptB and HptC with the same domains dropped below 28% (Figure 5B). Therefore, the sequence similarities and the close localization to the *cupC* operon suggested that PA2583 and HptA could be additional partners of the complex Roc system.

To test this hypothesis, we overproduced the full length of the PA2583 HK or its cytosolic part in a strain carrying a transcriptional fusion of the *cupC* promoter with *lacZ*, either alone or in combination with the overexpression of *hptA*. However, no stimulating effect on *cupC* expression was observed, even when the expression of the entire chromosomal *PA2583* gene was directly driven by a P*BAD* promoter (data not shown). However, we cannot exclude the possibility that the overproduced proteins are not active, since no control target was identified. Therefore, we investigated the possible interaction of PA2583 and HptA with different Roc proteins using the bacterial two‐hybrid system (Karimova et al. 1998) which has previously been used to study interactions in the Roc1 and Roc2 systems (Kulasekara et al. 2004; Sivaneson et al. 2011). While HptB interacted with its known partner SagS as expected (Hsu et al. 2008), we observed that HptA was able to interact with PA2583 and the D2 domains of three RRs, RocA1, RocA2, and RocR3 (Figure 5C), revealing the first identified partners for the HptA protein of *P. aeruginosa* . Therefore, based on sequence homology and interaction analyses, PA2583 was hereafter named as RocS4.

Considering the different genetic environments of *hptA* in PAO1, PA14, and IHMA87 strains, we further investigated the gene location in *P. aeruginosa* and *P. paraeruginosa* species (Figure 5D). In clades 3 and 4, except in strain RDJ20, *PA2583* was found in the vicinity of *hptA*, while it was absent in clades 1, 2, and 5 (Figure 5D). Moreover, the gene was consistently present in a region surrounded by two tRNA genes between *PA2584* and *PA2582*. The locus appears to be a hotspot for genetic rearrangements, as the distance between *PA2584* and *PA2582* varies greatly among strains, but *PA2583* was always found in the vicinity of one or the other gene (Figure S5). Notably, *PA2583* was predicted to be a pseudogene due to a frameshift in numerous clade 1 and 2 strains.

In conclusion, comparative genomics suggested a possible involvement of a hybrid kinase and the HptA protein in the signaling network of Roc TCSs. Interactions between the HK RocS4 and HptA, as well as between this Hpt protein and three RRs, were observed in vivo, suggesting the presence of an additional phosphorelay involving multiple RRs of the Roc system.

### 2.6 The Roc System Contributes to the Diversity of the Pangenomic Repertoire of RRs

**FIGURE 6:**
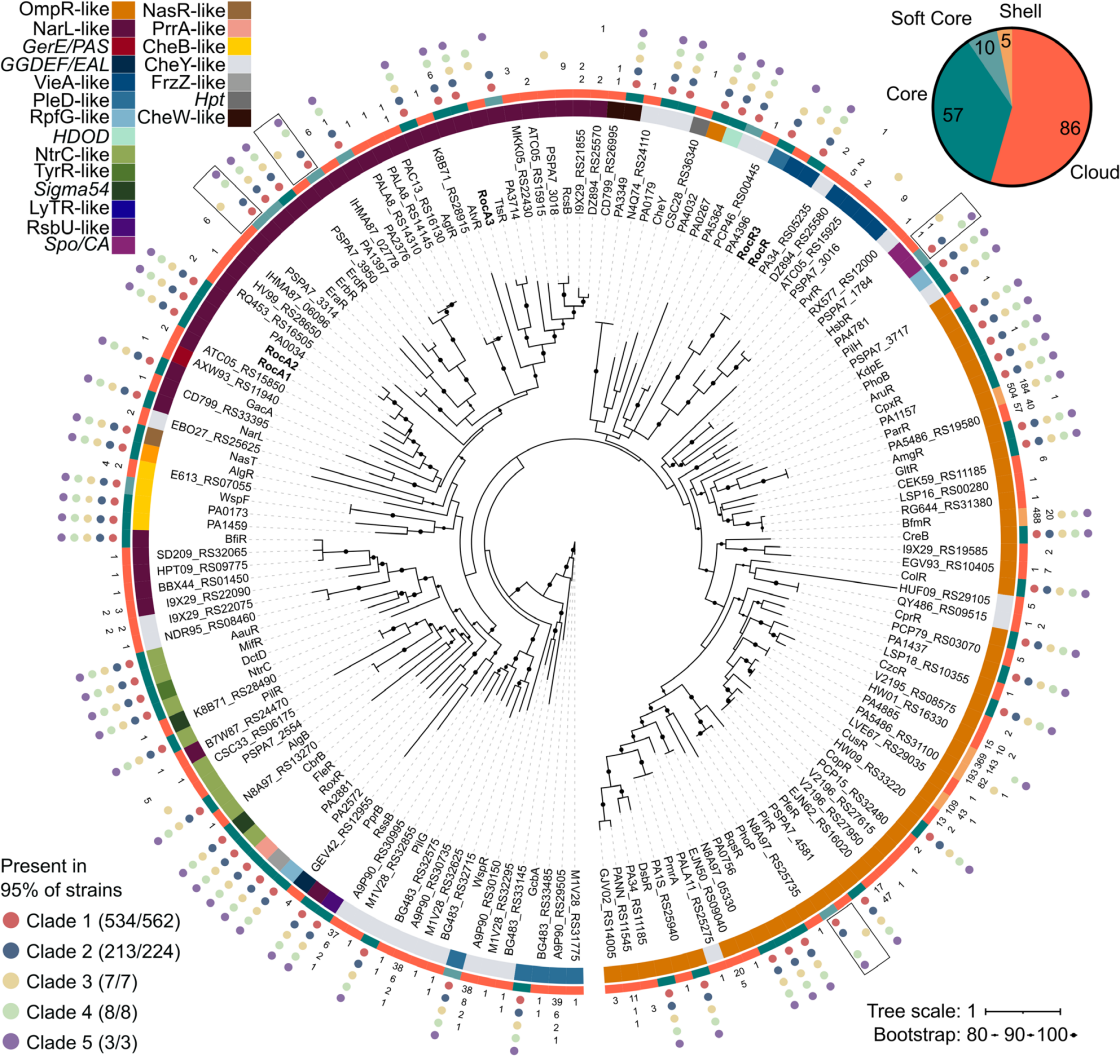
Roc players in the pangenomic repertoire of RRs in *Pseudomonas aeruginosa* and *Pseudomonas paraeruginosa*. The unrooted phylogeny of the REC domain of 158 identified RRs is shown in the center. Phylogenetic support is shown by black circles for nodes with high bootstrap value (> 80%). Labels correspond to the names of the proteins in PAO1, PA14, or PA7 where possible, otherwise the name of one representant was randomly chosen. Roc actors are highlighted in bold. RRs were classified into families based on their domain architecture, shown by the color in the first ring. The second ring represents whether the gene coding for the protein belongs to the core, soft core, shell or cloud genome of the species. The outer part of the tree shows the conservation of RRs in different clades. Raw counts are given except when more than 95% of the clade harbor the protein, in which case the values are replaced by a colored bubble. The 4 RRs with a specific distribution and separate homologs in clade 3 are shown in boxes.

Previous studies have established the phylogeny of the RRs present in the *P. aeruginosa* PAO1 strain but omitted certain RRs such as RocA3 (Chen et al. 2012). To integrate RocA3 into the RRs phylogeny and expand our comprehension of the distribution of RRs across the clade, we carried out a genome‐wide analysis of RRs at the level of *P. aeruginosa* and *P. paraeruginosa* species. Out of 804 predicted proteomes of the two species, 158 RRs were identified and classified into 21 families according to their domain architectures (Figure S6A) (Ortet et al. 2015). We then performed a phylogenetic analysis of the REC domain of the 158 RRs protein sequences (Figure 6).

Our results are broadly consistent with the previous analysis, which grouped the different families together as expected (Chen et al. 2004). Proteins with a single REC domain (CheY‐like) are found throughout the tree, illustrating the modularity of these proteins, which can lose domains by insertion or recombination. In *P. paraeruginosa*, four RRs have sequences that diverge below the similarity threshold (90% sequence identity) from their homologs in other clades (HsbR, PirR, EraR, and PA1397). RRs thus follow different evolutionary rates within the two species studied, suggesting that the redirection of RR functions occurs at different rates. The genome sizes of the strains from the 5 clades are similar with a mean size of 6.74 Mbp, although clade 3 strains, which correspond to the *P. paraeruginosa* species, have more RRs with an average of 75 RRs per strain compared to 70 for clades 1, 2, 4, and 5 (Figure S6B,C). By determining whether these RR‐encoding genes belong to the core (genes present in > 99% of the 804 strains), soft core (95%), shell (15%) or cloud genome of these species, we evaluated the pangenomic distribution of genes of the different RR families (Figure S6D). The most represented families (*ompR*‐like, *narL*‐like, and *cheY*‐like) show a broad distribution in the cloud genome, whereas smaller families show a more restricted distribution, some of them exclusively in the core genome (e.g., *nasR*‐like). The emergence of new RRs is often the result of duplication, and the restriction of these families to the core genome may be explained by an immediate disadvantage to the appearance of paralogs. The *vieA*‐like family is an exception: although poorly represented in the core genome with only *rocR*, genes encoding these RRs are the fourth most abundant in the cloud genome, suggesting that a variable distribution of these regulators between clades is common, as observed for *rocR3*. All strains studied have at least one RR whose gene does not belong to the core or soft core genomes, with clade 3 logically having the most RRs in the shell and cloud genomes (Figure S5E).

Concerning the RRs of the Roc system, the genes coding for RocA1, RocA2, RocA3, and RocR are part of the core genome. Within the *NarL*‐like family, RocA3 is phylogenetically distant from RocA1 and RocA2. The latter two are close and are similar to PA0034, which forms a TCS with LadS and regulates *cupA1* (Guo et al. 2024). Regarding RocA3, it is closely related to TtsR and PA3714, two homologs that were separated in our analysis because of their level of identity being below the threshold. This separation was observed for the 4 RRs discussed above, but presents a particular pattern for TtsR and PA3714 since TtsR is present in clades 3 and 4. In the strain PA7 of clade 3, TtsR was shown to regulate the expression of *txc*, a secretion system‐coding operon located in the RGP69 genomic island, which is inserted just downstream of this RR‐coding gene in clades 3, 4, and 5 (Cadoret et al. 2014). RocR3 is close to RocR and to a RR with only one REC domain (PA34_RS05235), which results from the insertion of a mobile genetic element into the *rocR* gene in two strains.

In conclusion, the RR regulatory network is largely conserved within *P. aeruginosa* and *P*. *paraeruginosa* species, which possess 57 core RRs. However, these conserved proteins represent less than half of the diversity of RRs observed at the pangenome scale, demonstrating the high plasticity of these networks. In this study, a clade‐specific distribution of RRs was rarely observed, and only a few RRs, such as PA4396, PSPA7_3016, PSPA7_2554, and RocR3, discriminate the RR composition of the different clades.

## 3 Discussion

**FIGURE 7:**
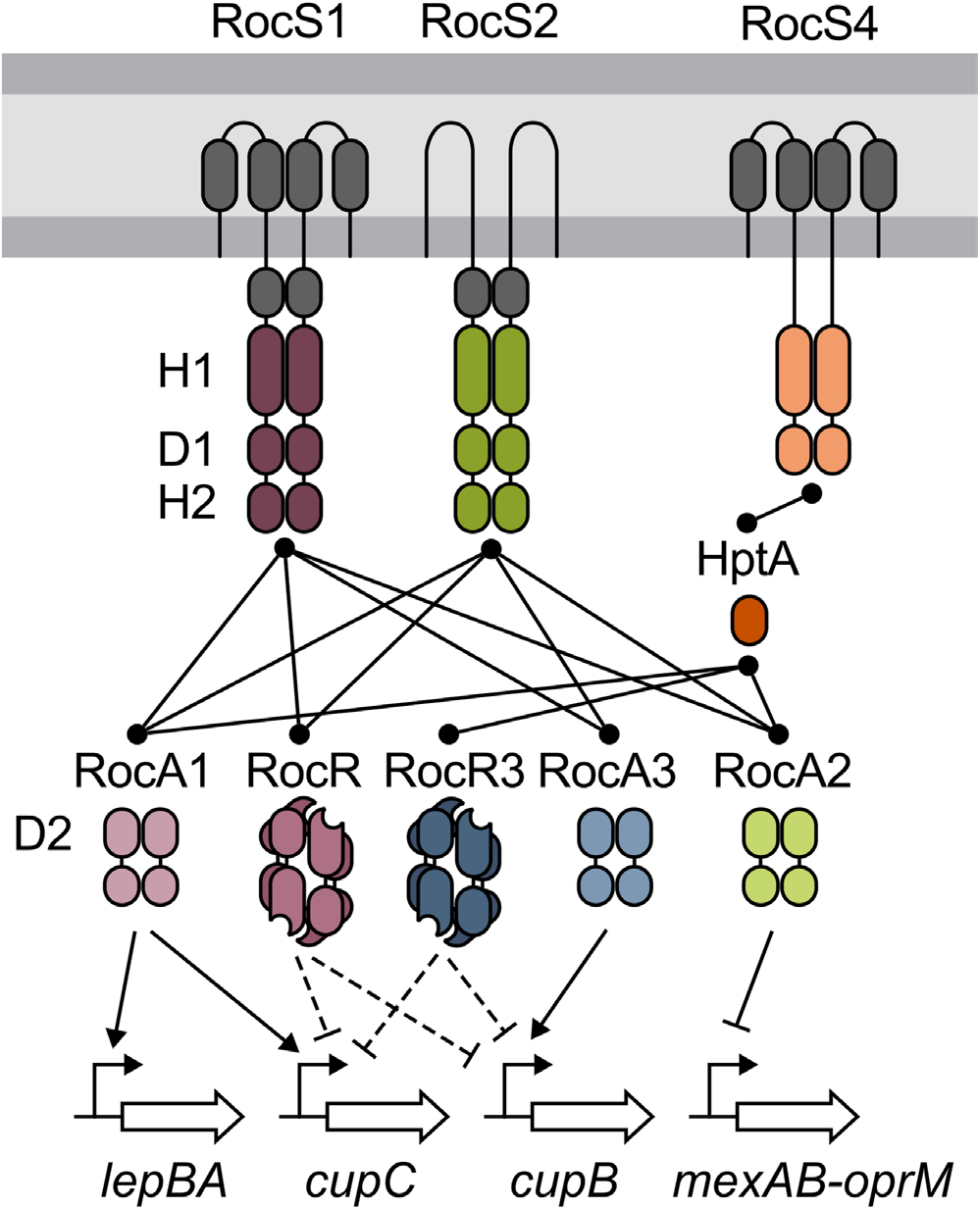
Schematic model of the Roc signaling pathways. RocA3 (PA4080), and the activation of the *lepBA* operon by RocA1 are added. The system was extended with potential new players: The phosphorelay consisting of HptA and the hybrid HK PA2583, named RocS4, and the EAL domain protein RocR3 found in a few *P. aeruginosa* and *P*. *paraeruginosa* strains. RocR3 is depicted with the same tetrameric structure as RocR (Chen et al. 2012). Domains shown are according to PFAM nomenclature; SBP_bac_3 (periplasmic grey domain), PAS_4 (cytosolic grey domain), HisKA and HATPase_c (H1), Response_reg (D1 and D2), Hpt (H2) and either GerE (DNA‐binding domain: Plain shape) or EAL (c‐di‐GMP phosphodiesterase; hollow shape) for output domain of RRs.

Although the Roc system is key to *P. aeruginosa* pathogenicity, our understanding of some of its critical regulatory aspects was still fragmented. This study combined experimental approaches with comparative genomics to unravel the interplay involved in the Roc system, completing the previous model and proposing new partners. Specifically, we characterize four novel players, including two RRs, a hybrid HK, and an Hpt protein, almost doubling the size of this interconnected network of TCSs. First, we identified PA4080, which we named RocA3, as a fourth RR capable of being activated by the unorthodox HKs RocS1 and RocS2 and of activating the expression of the *cupB* operon involved in biofilm formation. Although we suspected that RocA3 was the activator of *lepBA*, co‐regulating the genes of CupB5 and its transporter LepB, we found that RocA1 was the main transcriptional regulator of this operon. Comparative genomics allowed us to identify a different genetic organization of the *roc3* locus in *P. paraeruginosa* and in a specific clade of *P. aeruginosa* (clade 4), where a RocR homolog named RocR3 was encoded in the vicinity of *rocA3*. The regulators RocR and RocR3 are VieA‐like RRs, both capable of downregulating the expression of the *cupC* and *cupB* operons when overproduced in combination with RocS2. Analysis of the synteny of the *cupC* locus led us to study the hybrid HK PA2583 and the Hpt protein HptA, which showed strong sequence homologies with different domains of RocS1 and RocS2. Genomic comparison revealed that *hptA* is located in close proximity to the RocA1‐controlled *cupC* operon in all strains studied. Interestingly, in *P. paraeruginosa* and a specific clade of *P. aeruginosa* (clade 4), *PA2583* is adjacent to *hptA*, whereas the gene is located in a different locus surrounded by two tRNAs genes in other clades of *P. aeruginosa* . The D1 domain of PA2583 interacts with HptA, which in turn interacts with the D2 domains of RocA1 and RocA2. This is the first report of partners for HptA, which appears to be able to link different RRs of the Roc system to the unorthodox HK PA2583, which we called RocS4 because of its probable implication in the Roc system. The HptA protein was also able to interact with RocR3, highlighting the potential integration of this clade‐specific putative RR in the Roc system. Finally, we delineated the repertoire of RR‐encoding genes across the pangenome of *P. aeruginosa* and *P. paraeruginosa* and mapped the conservation of these regulators, which can be variable across strains, as shown for the Roc system. Overall, our results have expanded our understanding of the Roc system, proposing a new comprehensive view (Figure 7) by discovering conserved or clade‐specific partners that form a plastic and complex network of TCSs that regulate pathogenicity.

In Roc signaling pathways, the HKs RocS1 and RocS2 are able to interact and activate multiple RRs, whose genes are located in different loci. These HKs possess different sensory domains, potentially offering the integration of different incoming signals. Indeed, as predicted for the hybrid HK PA2583, RocS1 possesses two consecutive solute binding protein (SBP) domains belonging to the SBP_bac_3 (PF00497) family, which are known to frequently detect amino acids (Matilla et al. 2021). In addition, the three HKs possess a cytoplasmic PAS (Per‐Arnt‐Sim) domain that could be involved in protein dimerization, ligand binding, or oxygen/redox sensing (Henry and Crosson 2011). Because the Roc system is able to reduce the antibiotic resistance of the bacteria while activating their biofilm formation, it was suggested that it may be important for adaptation to the lungs of cystic fibrosis (CF) patients (Sivaneson et al. 2011). The signals perceived by HKs are rarely known, and the study of TCSs often involves their artificial activation, which can be achieved by overexpressing the RR or HK genes, as was done in this work. However, this is not always effective, as observed with PA2583, and predicting the outcome of such overexpression is difficult. Indeed, competition between different TCS partners involves finely tuned concentrations of HKs and RRs for a proper signaling. For example, HKs are usually less numerous than RRs to favor out‐competition within RRs and reduce cross‐talk with noncognate RRs (Laub and Goulian 2007; Siryaporn and Goulian 2008). In addition, phosphotransfer efficiency can differ between an HK and its multiple partners, as shown for the asymmetric cross‐talk between NarXL and NarQP, with NarX interacting preferentially with NarL (Noriega et al. 2010). Finally, HKs can be bifunctional, controlling both phosphorylation and dephosphorylation of cognate RR(s). Therefore, artificial activation of a system may lead to hazardous results that must be distinguished from natural in vivo activation. In the Roc system, we do not know if RocS1 and RocS2 are bifunctional HKs. Moreover, their overproduction activates the signaling pathways only in M63 medium and not in LB, suggesting a potential lock that is lifted in the favorable medium. Only the activation of the HKs by their physiological signals could allow the normal functioning of the systems, integrating all the dynamics of the different partners and allowing us to determine the exact extent of cross‐talk and cross‐regulation.

Why should bacteria maintain all the interconnected TCSs of the Roc system? When TCSs are duplicated, the proteins usually need to rapidly gain new functions in order to be maintained through different mechanisms, such as changes in HK sensory domains and pathway inputs or changes in RRs and pathway outputs (Capra and Laub 2012). As mentioned above, RocS1 has specific periplasmic sensory domains that its closest paralog RocS2 does not possess, suggesting that they have undergone rearrangement of their sensory domains. Furthermore, although the identified binding sites of RocA1, RocA2, and RocA3 present similarities, they control different targets in vivo, indicating that their transcriptional activity differs (binding capability and/or interaction with RNA polymerase). In conclusion, although communication within the network formed by the Roc partners appears to be interconnected, it seems that the signal detection domains of the HKs and the effector domains of the RRs are very specific to each component, allowing multiple genes to be controlled in response to different potential signals. The rationale behind the interconnection of TCSs remains elusive, the simplest explanation being the ability to integrate multiple stimuli to regulate a large number of genes. Such a network needs to be considered in the light of physiological activation implementing, for example, competition between RRs and feedback regulatory loops, as seen here with RocA3, making the Roc system probably a more subtle system than just a relay between multiple signals and multiple regulators.

Our RRs phylogeny raises questions about the Roc system and its variability, which seem to escape every simple evolutionary scenario. The phylogenetic distance observed between RocA3 and RocA1/RocA2 is unexpected in two respects. Functionally, our study shows that RocA3 is integrated into the Roc system, demonstrating that, despite differences, this RR is able to communicate with RocS1 and RocS2. In addition, RocR3, whose gene is located in the same locus as *rocA3* in some strains, is phylogenetically very close to RocR, suggesting a duplication. Furthermore, the polymorphisms observed at the *roc3* and *hptA* loci are surprisingly incongruent with the established species tree in the way that clades 1, 2, and 5 are grouped together. The explanation for this discrepancy is not clear, since the genetic scars left by the putative loss of *rocR3* and *PA2583*, respectively, are similar in these clades, tending to exclude the parallel loss and rearrangement of the genes (Figure S4). It is then tempting to assume that the maintenance of the ancestral polymorphism observed in the intermediate clade 4 for both loci arose from deep coalescence, providing a new perspective on our understanding of the evolution of *P. aeruginosa* and *P. paraeruginosa* species. Finally, the reason for these different organizations remains elusive, and one can only speculate on the functioning of the system in these clades based on our observation for the PAO1 strain.

In conclusion, TCS conservation within species is variable and, as we showed in *P. aeruginosa* and *P. paraeruginosa*, the distribution and organisation of the genes encoding these regulatory proteins are important determinants of the strain‐dependent diversity of TCS regulatory networks (Trouillon et al. 2021; Elsen et al. 2024). Our work showed how a one‐of‐a‐kind TCS network, mostly conserved in *P. aeruginosa* and *P. paraeruginosa* species, was differentially shaped in these species by the loss and recruitment of different players.

## 4 Materials and Methods

### 4.1 Bacterial Strains and Growth Conditions

The *Escherichia coli* and *Pseudomonas aeruginosa* strains used in this study are described in Table S1. Bacteria were grown aerobically in lysogeny broth (LB) or in M63 minimal medium (100 mM KH_2_PO_4_, 15 mM (NH_4_)_2_SO_4_, 1.7 μM FeSO_4_, 1 mM MgSO_4_, 0.2% glucose, 0.5% casamino acids, pH 7) at 37°C. *P. aeruginosa* was also cultured on Pseudomonas Isolation Agar plates (PIA Difco). To assess the β‐galactosidase activities of strains carrying chromosome‐integrated *lacZ* fusions, LB‐grown cells were diluted to an OD_600_ of 0.1 in M63 medium with the appropriate antibiotics and inducers (0.5% arabinose for pJN105‐derived plasmids used alone, and 0.2% when combined with a pMMB‐derived plasmid; 10 μM IPTG for pMMB‐RocS1, and 1 mM IPTG for pMMB‐RocS2) when required and incubated with shaking for 6 h before assays were carried out. Antibiotics were added at the following concentrations (in μg/ml): 100 ampicillin (Ap), 10 chloramphenicol (Cm), 50 gentamicin (Gm), 10 tetracycline (Tc) for *E. coli* ; 300 carbenicillin (Cb), 200 Gm, 200 Tc for *P. aeruginosa* .

### 4.2 Plasmids and Genetic Manipulation

The plasmids utilized in this study and the primers used for PCR are listed in Tables S1 and S2, respectively. All the constructions were verified by sequencing.

To generate *P. aeruginosa* deletion mutants, upstream (sF1/sR1) and downstream (sF2/sR2) flanking regions of the different genes were fused and cloned into the *Sma*I‐cut pEXG2 plasmid by sequence‐ and ligation‐independent cloning (SLIC) using the appropriate primer pairs (Li and Elledge 2007). The same strategy was used to create the point mutation within the *rocA3* sequence, with the overlapping primers creating the mutation. To create the transcriptional *lacZ* fusions, fragments comprising the ATG sequence and around 500 bp upstream of each analysed target were amplified using the appropriate pairs (sF/sR) and inserted into *Sma*I‐cut miniCTX‐T*rrnB*‐*lacZ* by SLIC. To express genes under the P*lac* or the P*BAD* promoter, sequences containing the entire coding sequences with 50 bp upstream were amplified using the appropriate pairs (sF/sR) and inserted by SLIC into *Sma*I‐cut pBBR1MCS4 or *Sma*I‐cut pJN105, respectively. To produce PA2583/RocS4 in pMMB, pMMB‐RocS2 was modified by replacing the *rocS2* fragment, removed with *Bsr*GI and *Eco*RI, with *rocS4* fragments, prepared for pBBR‐RocS4, inserted by SLIC. For the plasmids used in the bacterial two‐hybrid systems, the sequences were amplified using the appropriate pairs (sF/sR) and inserted into *Bam*HI‐cut pKT25 or pUT18c by SLIC: this created hybrid proteins with heterologous proteins fused to the C‐terminal of T25 or T18 fragment of the *Bordetella pertussis* adenylate cyclase, respectively. For chromosomal insertion of the P*BAD* promoter upstream of the *PA2583*/*rocS4* gene, a pEXG2‐derived plasmid with upstream and downstream sequences of the *PA2583*/*rocS4* promoter was generated by SLIC, creating a *Spe*I site 50 bp upstream of the start codon. Then, the 1580 bp “*araC*‐P*BAD*” fragment was excised from pSW196 using *Xba*I and cloned into the created *Spe*I site to produce pEXG2‐P*BAD*‐*PA2583*‐Sp.

The pBBR‐, pJN105‐, and pMMB‐derived plasmids were introduced into *P. aeruginosa* by transformation, while the pEXG2‐ and miniCTX‐T*rrnB*‐*lacZ*‐derived vectors were transferred into *P. aeruginosa* strains by triparental mating using the helper plasmid pRK600. Allelic exchanges for mutagenesis were selected as previously described (Berry et al. 2018). To excise the miniCTX backbone of strains carrying the *lacZ* fusions, the pFLP2 plasmid was first introduced into the cells and, in a second step, the bacteria were streaked on medium containing 5% sucrose to select for the loss of pFLP2. To create the mutant encoding the RocA3_D58A_ protein, the mutated sequence was introduced into the Δ*rocA3* mutants to replace the deleted gene.

### 4.3 β‐Galactosidase Activity Assay

β‐Galactosidase activity was assayed as previously described (Miller 1977; Thibault et al. 2009), at least in triplicate. Activities were expressed in Miller Units (MU) and the error bars in the graphs indicate the standard error of the mean (SEM). When relevant, statistical significance was assessed using the ANOVA test with Tukey's or Dunnett's method. Numerical data and statistics underlying the graphs are provided in Table S3.

### 4.4 Bacterial Two‐Hybrid Assay

This assay was conducted as previously described (Kulasekara et al. 2004). The nomenclature used to define the proteins was as follows: the transmitter domain of the HK carrying the conserved histidine residue was called H1. Unorthodox HKs possess additional domains called D1 and H2. The receiver domain of the RR carrying the conserved aspartate residue was the D2 domain. The plasmids pKT25‐HptB and pUT18c‐SagS‐D1 were used as positive controls. The pKT25‐ and pUT18c‐derived plasmids were co‐transformed into the DHM1 strain using heat shock, and single colonies were patched on LB containing 5‐bromo‐4‐chloro‐3‐indolyl‐beta‐D‐galactopyranoside (X‐gal) at 40 μg/mL and 1 mM IPTG. Positive interactions were identified as blue colonies on X‐gal after a 24 h incubation at 30°C. Colonies were resuspended in water for β‐galactosidase assays.

### 4.5 DAP‐Seq Analysis

Published DAP‐seq data were collected with GEO Series accession number GSE179001 (Trouillon et al. 2021). DNA‐binding motif discovery was performed using MEME‐ChIP (Machanick and Bailey 2011). Relative fold‐enrichments were calculated for each RR on each genome. The PA14 dataset was used based on quality thresholds because the PA4080 experiment with the PAO1 genome did not provide sufficient DNA enrichment.

### 4.6 Phylogenetic Analyses

Phylogenetic analyses of *P. aeruginosa* and *P. paraeruginosa* were performed on 804 annotated genomes available in NCBI and Pseudomonas.com databases assessed in April 2024. The genomes used in this study are described in Table S4. Core gene alignments were obtained from Roary (minimum 90% identity and a presence in 99% of isolates to be considered core) (Page et al. 2015), and SNPs were extracted using SNP‐sites (Page et al. 2016). For phylogenetic analyses of RRs, 158 RRs were identified by determining the domain architecture of the proteins in the pangenome obtained from Roary with InterProScan (Jones et al. 2014). The genome‐wide repertoire of *P. aeruginosa* and *P. paraeruginosa* RRs is described in Table S5. REC domains were aligned with MAFFT using the L‐INS‐I option (Katoh and Standley 2013). Maximum‐likelihood trees were built using FASTTREE for genomes (Price et al. 2010) with default parameters and IQ‐tree with 1000 bootstrap replicates for REC domains (Nguyen et al. 2015), then visualized and annotated using iTOL (Letunic and Bork 2021).

### 4.7 Bioinformatic Analyses

Pangenomic distributions of genes were obtained from Roary. Sequence alignment and visualization were performed using Clinker (Gilchrist and Chooi 2021). Sequence homologies were evaluated with Clustal Omega (Madeira et al. 2022). Variation in genetic regions was examined using Jalview (Waterhouse et al. 2009). Most tools were used on the European or American Galaxy servers (The Galaxy Community 2022).

## Author Contributions

**Victor Simon:** conceptualization, investigation, formal analysis, writing – original draft, writing – review and editing. **Julian Trouillon:** formal analysis, writing – review and editing. **Ina Attrée:** writing – review and editing. **Sylvie Elsen:** conceptualization, investigation, writing – original draft, writing – review and editing.

## Conflicts of Interest

The authors declare no conflicts of interest.

## Supporting information


Data S1.

Data S2.

## Data Availability

The data that support the findings of this study are available in the Supporting Information S1 and S2 of this article.
