# Supplementary material for: Functional and Pangenomic Exploration of Roc Two‐Component Regulatory Systems Identifies Novel Players Across Pseudomonas Species
Source: Mol Microbiol. 2025 Mar 14;123(5):439–53. doi: 10.1111/mmi.15357 (PMC12051241; doi:10.1111/mmi.15357)
Supplement: Supplementary file 1 — Data S1. [file MMI-123-439-s002.pdf]

Supplementary Information

Functional and pangenomic exploration of Roc two-component regulatory systems identifies novel players across *Pseudomonas* species

Victor Simon[1,2#], Julian Trouillon[3], Ina Attrée[1] and Sylvie Elsen[1#]

[1]University Grenoble Alpes, Institute of Structural Biology, UMR5075, Team Bacterial Pathogenesis and Cellular Responses, 38054 Grenoble, France

[2]Present address: Université de Lyon, INSA Lyon, Université Claude Bernard Lyon 1, CNRS UMR5240, Laboratoire de Microbiologie, Adaptation et Pathogénie, 69621 Villeurbanne, France.

[3]Institute of Molecular Systems Biology, ETH Zürich, 8093 Zürich, Switzerland

[#]co-corresponding authors: victor.simon@insa-lyon.fr; sylvie.elsen@ibs.fr

Supplementary Figure S1

Supplementary Figure S2

Supplementary Figure S3

Supplementary Figure S4

Supplementary Figure S5

Supplementary Figure S6

Supplementary Table S1: Bacterial strains and plasmids used in this work

Supplementary Table S2: Primers used in this work

Supplementary Table S3: Numerical data and statistics underlying graphs

Supplementary Table S4: Genomes dataset used in this work

Supplementary Table S5: Genome-wide repertoire of *Pseudomonas aeruginosa* and *Pseudomonas paraeruginosa* response regulators

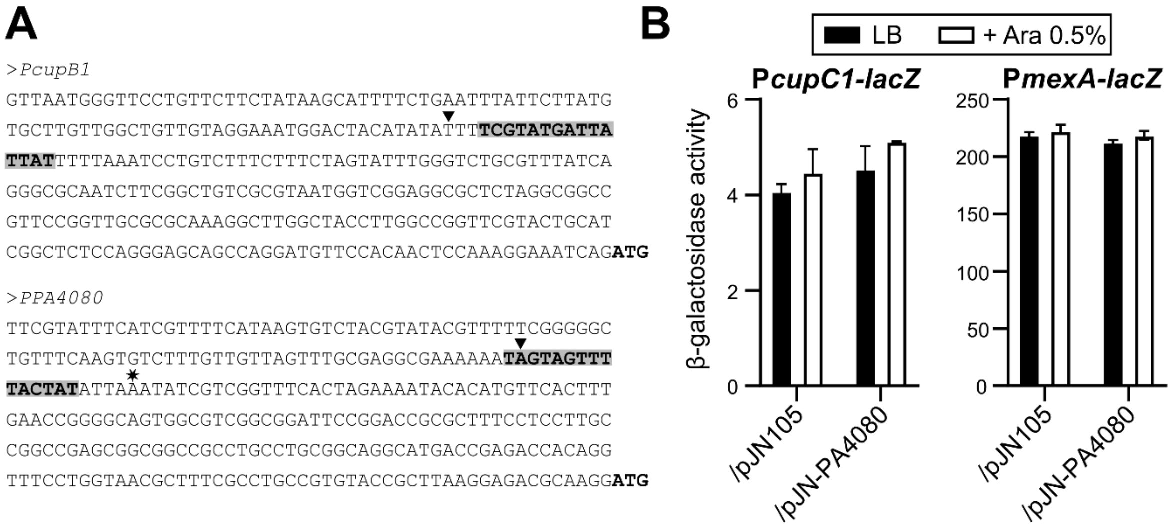

FIG S1. PA4080 regulates the *cupB* operon, its own expression but not other Roc targets (A) Upstream sequences of the *cupB1* and *PA4080* genes. The ATG of each coding sequence is indicated as well as the location of the transcriptional start site of *PA4080* (star). The predicted binding site for PA4080 on each sequence is shown in bold and coloured in grey, with the top of the DAP-seq peak (Trouillon *et al.*, 2021) indicated by an arrowhead. (B) β-galactosidase activities of the indicated strains carrying the P*cupC1-lacZ* or P*mexA-lacZ* transcriptional fusions. The strains also carried either the empty pJN105 or the pJN-PA4080 plasmid, and expression of *PA4080* was induced with 0.5% arabinose for 2.5 h in LB medium. Experiments were performed in triplicate and the error bars represent the SEM. Statistical analysis was performed using two-way ANOVA, followed by Dunnett's test for comparison to the control condition (PAO1 WT /pJN105 in LB). No statistically significant differences were detected ($p > 0.05$).

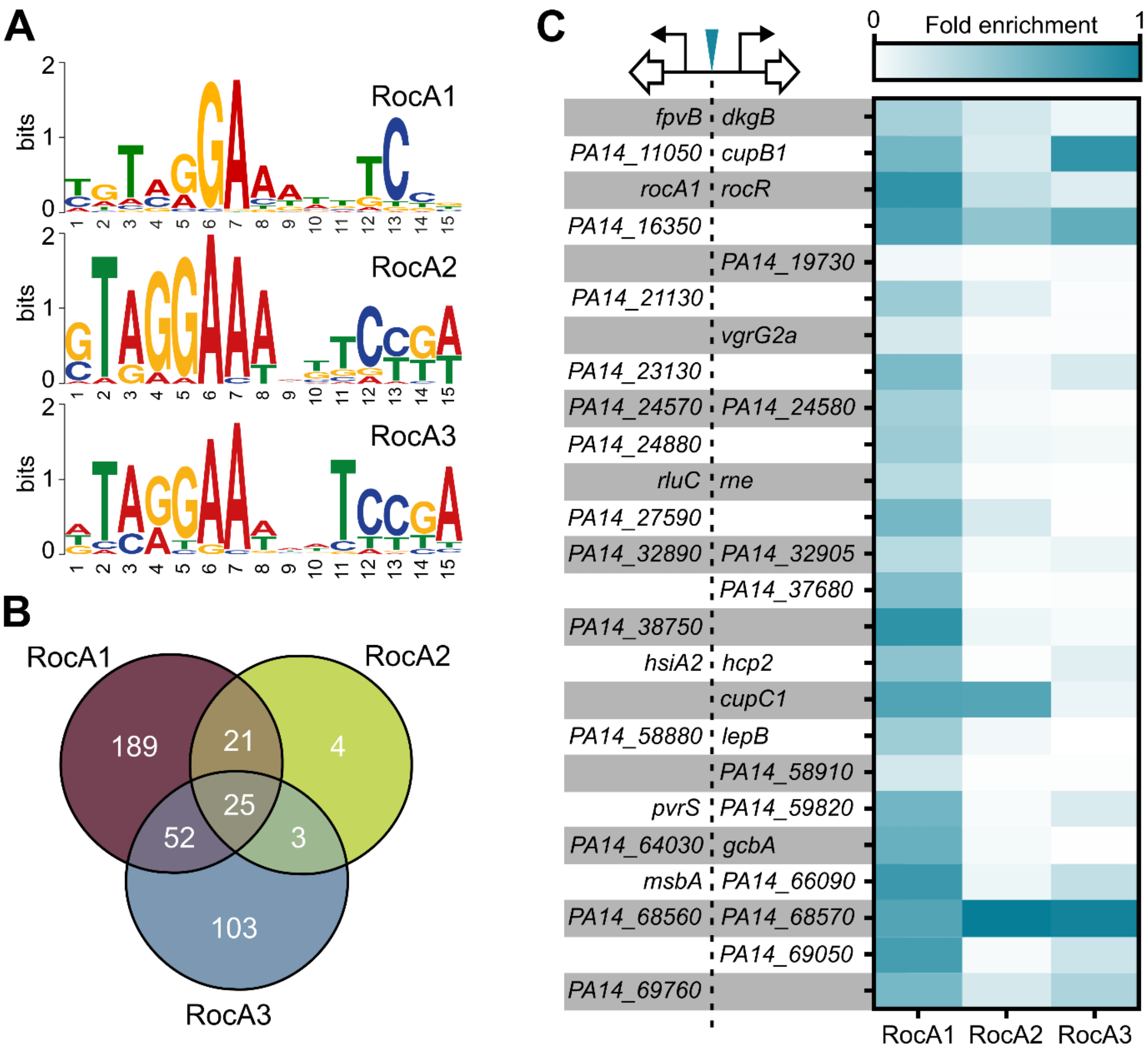

Fig S2. RocA1, RocA2 and RocA3 targets on the PA14 genome. Reanalysis of the dataset of DAP-seq results from published data (Trouillon *et al*., 2021) (A) DNA-binding motifs of RocA1, RocA2 and RocA3 identified by MEME-ChIP. The amino acid symbols are shown at each position. Sequence conservation at each position is indicated by the total height of the stack. The relative frequency of each amino acid is indicated by the height of the symbols within the stack. (B) Overlap of the inferred targets of RocA1, RocA2 and RocA3. (C) Relative fold-enrichment of 25 common targets of RocA1, RocA2 and RocA3. Intergenic target regions are represented on the left, indicating the first gene of potentially regulated transcription units.

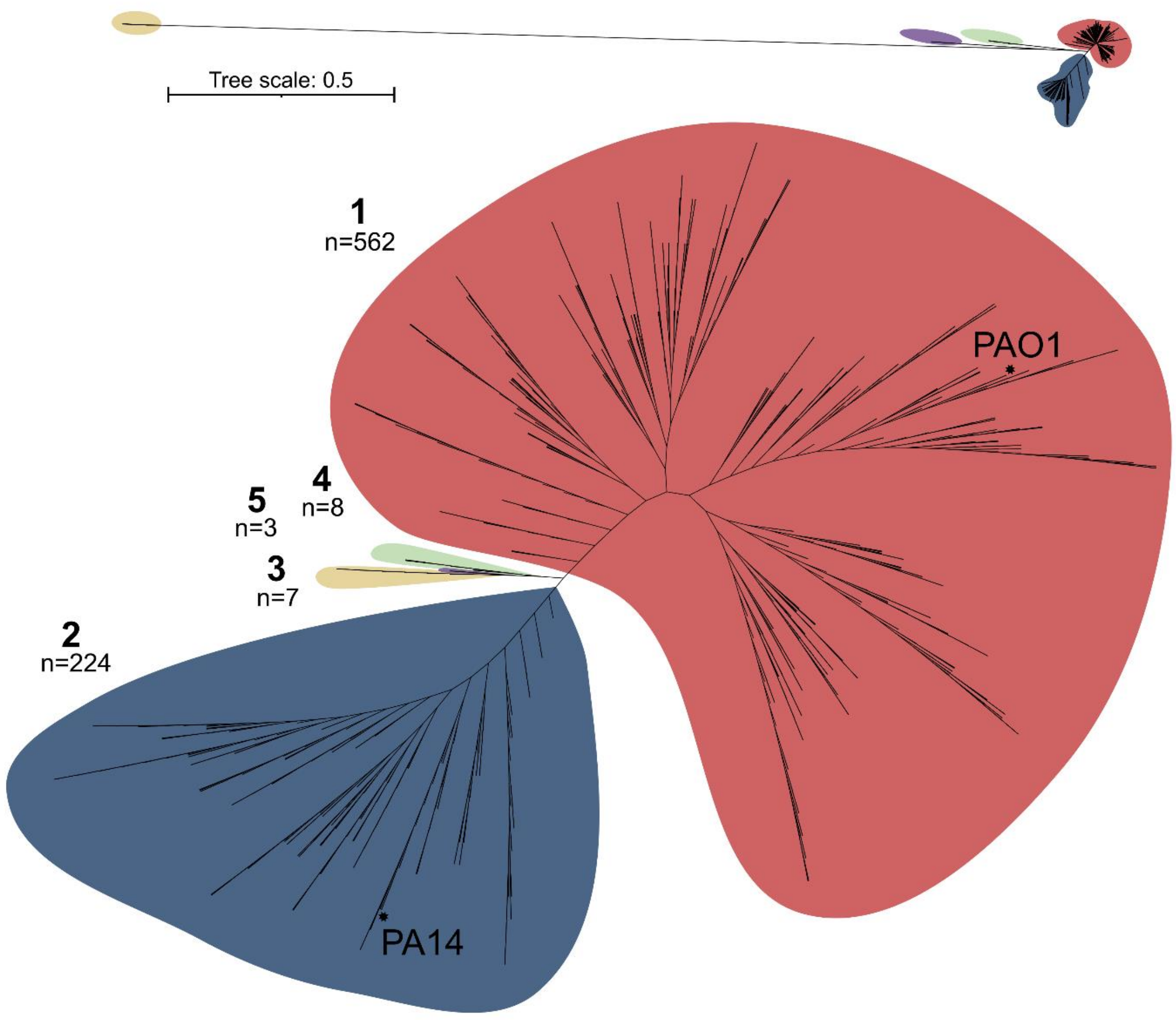

FIG S3. Unrooted phylogenetic tree of *P. aeruginosa* and *P. paraeruginosa* species. The number of genomes for each clade is given (n=804) and the positions of the PAO1 and PA14 reference strains are indicated by a star. The small tree in the upper part represents actual genetic distances between the clades, as indicated by the scale.

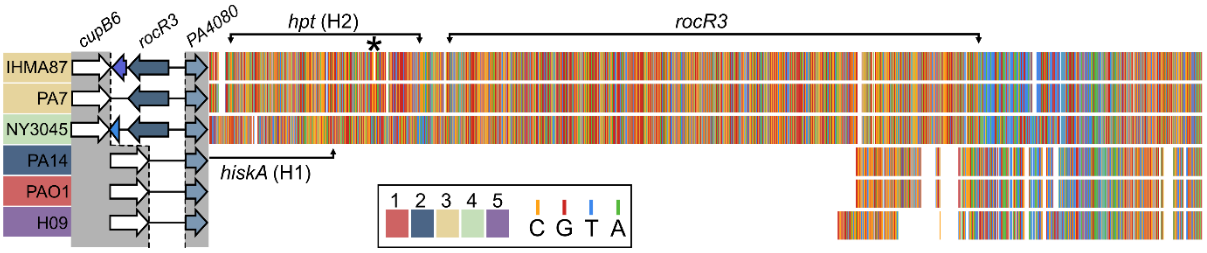

Fig S4. Genetic variations in the *roc3* locus. Alignment of 6 sequences of the genetic region between *rocA3* and *cupB6* for six strains from different clades. The position of different CDSs is highlighted by brackets: the Hpt-encoding gene (*hpt* or H2 domain), the HiskA-encoding gene (*hisKA* or H1 domain), and *rocR3*. The asterisk indicates the 4-bp deletion in IHMA87.

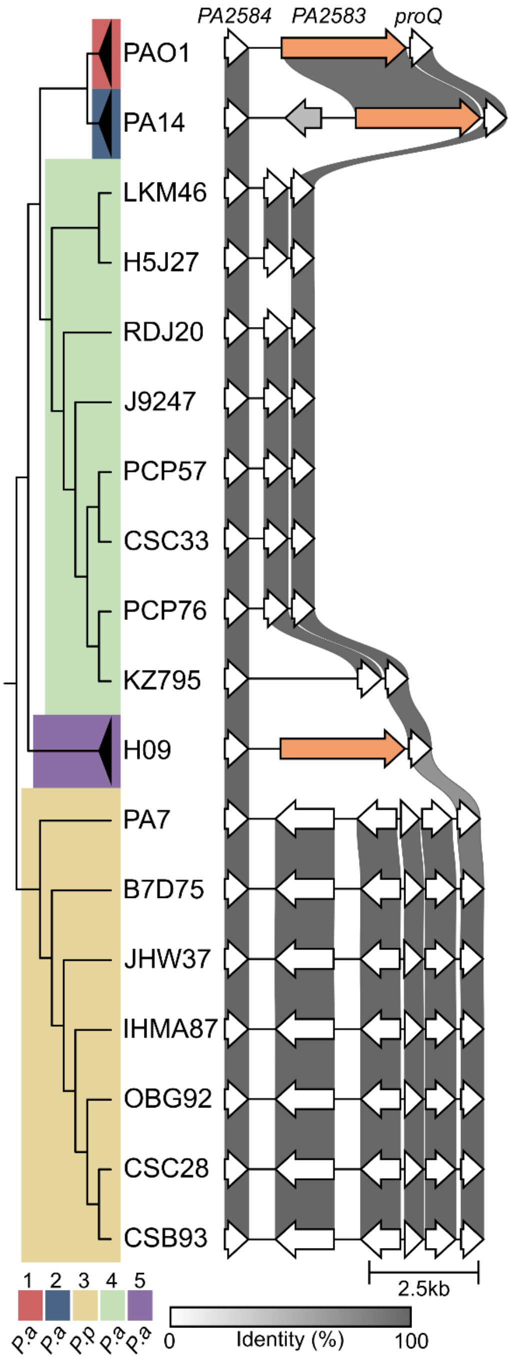

Fig S5. The *PA2583* locus in *Pseudomonas aeruginosa and Pseudomonas paraeruginosa* species. Genetic comparison of the *PA2583* locus in clade 3 and 4 strains, with the percentage of sequence identity indicated by the grey scale. PAO1, PA14 and H09 were used as reference for clades 1, 2 and 5, respectively. Sequences were ordered based on the genomic phylogeny shown.

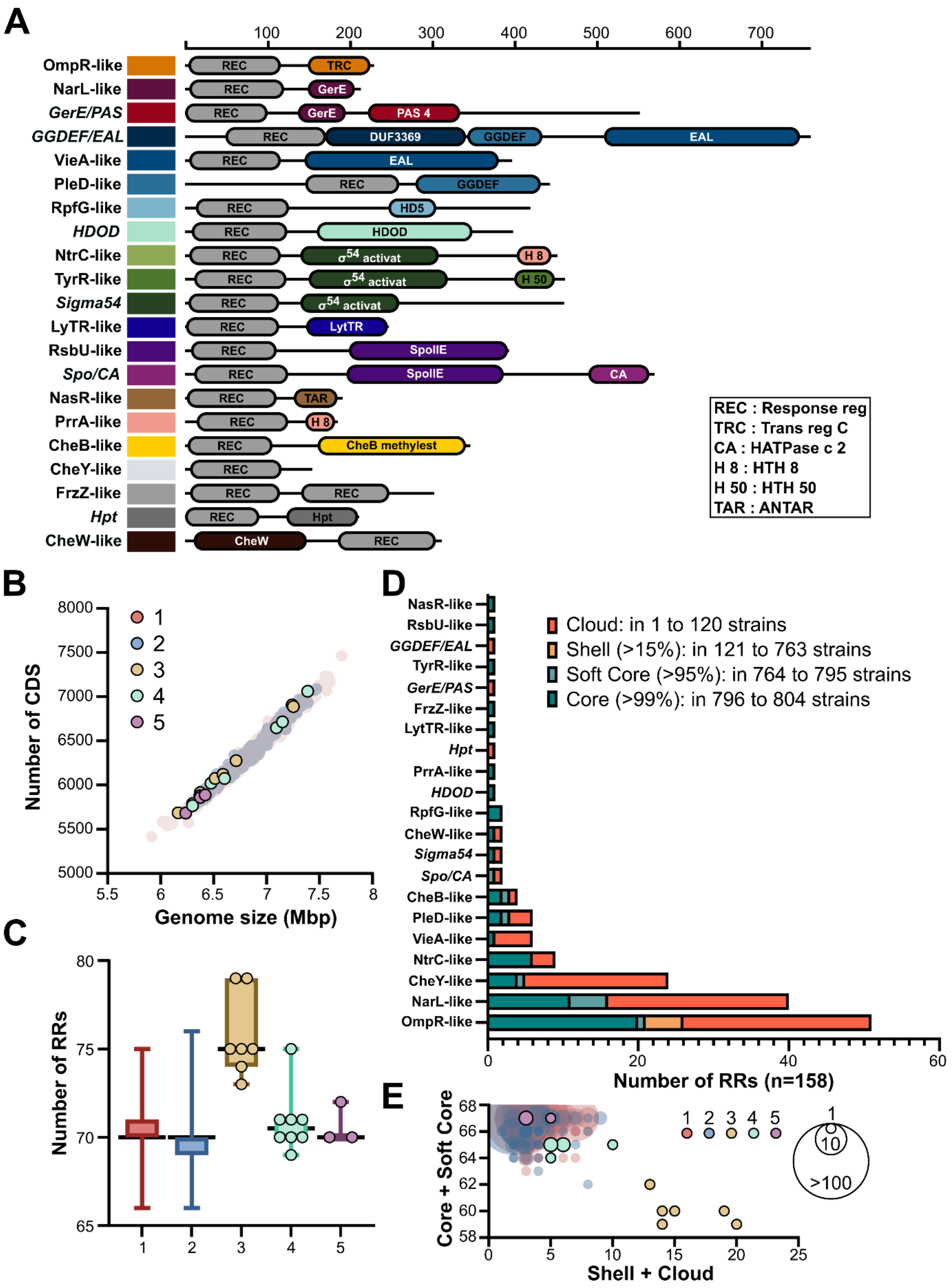

Fig S6. Pangenomic repertoire of RRs in *Pseudomonas aeruginosa* and *Pseudomonas paraeruginosa*.

(A) Domain architectures of the different RRs families identified using Pfam names. The amino acid length of the domains corresponds to the average of the different representatives for each family. The

names of the families are chosen according to previous work (Ortet et al., 2015), except for six families whose names, in italics, were given based on the domain associated with the REC domain. (B) Genome sizes and total number of predicted CDSs for the 804 genomes of *P. aeruginosa* and *P. paraeruginosa* classified by clades. Superabundant representatives of clades 1 and 2 are shown as transparent. (C) Number of RRs identified in each genome classified by clade. The boxes represent the mean, maximum, and minimum values, as well as the 95% interval of the distribution. Individual values are shown for clades 3, 4 and 5. (D) Distribution of different RRs in the core, soft core, shell and cloud genomes classified into families. (E) Distribution between core/soft core RRs and shell/cloud RRs according to different genomes. The size of the bubbles reflects the number of genomes with identical values. Clades 1 and 2 are shown as transparent.

Table S1. Bacterial strains and plasmids used in this work

Strain or plasmid	Genotype or relevant properties	Reference/Source
**Strains**		
P. aeruginosa		
PAO1	Wound isolate, sequenced laboratory strain	J. Mougous
PAO1Δ*PA4080*	PAO1 with *PA4080* (*rocA3*) deletion	This study
PAO1Δ*rocA1*	PAO1 with *PA3948* (*rocA1*) deletion	This study
PAO1Δ*rocA2*	PAO1 with *PA3045* (*rocA2*) deletion	This study
PAO1Δ*rocA1*Δ*rocA2*	PAO1 with *rocA1* and *rocA2* deletion	This study
PAO1Δ*rocA1*Δ*rocA2*Δ*rocA3*	PAO1 with *rocA1*, *rocA2* and *rocA3* deletion	This study
PAO1-*PA4080 D58A*	PAO1 with *rocA3* mutated gene encoding $RocA3_{D58A}$	This study
PAO1 :: P*BAD-PA2583*	PAO1 with *PA2583* (*rocS4*) under the control of *in situ* inserted P*BAD* promoter	This study
P. paraeruginosa		
IHMA879472 (IHMA87)	wild-type strain (urinary infection)	(Kos *et al.*, 2015)
E. coli		
TOP10	Chemically competent cell	Invitrogen
DHM1	F^- *cya-854 recA1 endA1 gyrA96* (Nal^R) *thi1 hsdR17 spoT1 rfbD1 glnV44* (AS)	(Karimova *et al.*, 1998)
**Plasmids**		
pRK600	Helper plasmid with conjugative properties (Cm^R)	(Kessler *et al.*, 1992)
pFLP2	Plasmid expressing the Flp recombinase (Ap^R/Cb^R)	(Hoang *et al.*, 1998)
pMMB67EH-Gm	Broad-host-range bacterial cloning vector, *tac* promoter (Gm^R)	This study
pMMB67-RocS1	pMMB67EH-GW harbouring the *rocS1* gene from Gateway library (Gm^R)	(Kulasekara *et al.*, 2005)
pMMB67EH	Broad host range bacterial cloning vector, P*tac* promoter (Ap^R/Cb^R)	(Sivaneson *et al.*, 2011)
pMMB67-RocS2	pMMB67HE harbouring the *rocS2* gene from Gateway library (Ap^R/Cb^R)	(Sivaneson *et al.*, 2011)
pMMB-RocS4	pMMB67-RocS2 derived vector with *rocS4* replacing *rocS2* (*Bsr*GI-*Eco*RI - SLIC)	This study
pBBR1MCS4	Broad host range bacterial cloning vector, P*lac* promoter (Ap^R/Cb^R)	(Kovach *et al.*, 1995)
pBBR-RocS4	pBBR1MCS4 carrying SLIC fragments for *rocS4* expression	This study
pBBR-RocS4c	pBBR1MCS4 carrying SLIC fragments for expression of the	This study

	cytoplasmic portion of RocS4 (176-973 aa)	
pEXG2	Allelic exchange vector (Gm^R)	(Riestch *et al.*, 2005)
pEXG2 PAO1Δ*rocA1*	pEXG2 carrying SLIC fragment for *rocA1* deletion (Gm^R)	This study
pEXG2 PAO1Δ*rocA2*	pEXG2 carrying SLIC fragment for *rocA2* deletion (Gm^R)	This study
pEXG2 *PA4080-D58A*	pEXG2 carrying SLIC fragment for *rocA3* mutation (Gm^R)	This study
pEXG2-*PA2583*-Sp	pEXG2 carrying SLIC fragment for *araC*-P*BAD* insertion upstream of *PA2583*/*rocS4* (Gm^R)	This study
pEXG2-P*BAD*-*PA2583*-Sp	pEXG2-*PA2583*-Sp with *araC*-P*BAD* fragment from pSW196 (Gm^R)	This study
pSW196	Site-specific integrative plasmid with P*BAD30*-promoter (*attP* site, FRT, Tc^R)	(Baynham *et al*., 2006)
pJN105	Broad host range bacterial cloning vector, *araC*-P*BAD* promoter (Gm^R)	(Newman and Fuqua, 1999)
pJN105-PA4080	pJN105 carrying SLIC fragment for *rocA3* expression (Gm^R)	This study
pJN105-RocR	pJN105 carrying SLIC fragment for *rocR* expression (Gm^R)	This study
pJN105-RocR3	pJN105 carrying SLIC fragment for *rocR3* expression (Gm^R)	This study
pJN105-HptA	pJN105 carrying SLIC fragment for *hptA* expression (Gm^R)	This study
miniCTX-T*rrnB*-lacZ	Site-specific integrative plasmid with promoter less-*lacZ* and strong *rrnB* terminator (*attP* site, FRT, Tc^R)	(Elsen *et al.,*2024)
pCTXter-P*cupB1-lacZ*	miniCTX-T*rrnB-lacZ* harboring the *cupB1* promoter fused to *lacZ* (*attP* site, FRT, Tc^R)	This study
pCTXter-P*cupC1-lacZ*	miniCTX-T*rrnB-lacZ* harboring the *cupC1* promoter fused to *lacZ* (*attP* site, FRT, Tc^R)	This study
pCTXter-P*PA4080-lacZ*	miniCTX-T*rrnB-lacZ* harboring the *rocA3* promoter fused to *lacZ* (*attP* site, FRT, Tc^R)	This study
pCTXter-P*mexA-lacZ*	miniCTX-T*rrnB-lacZ* harboring the *mexA* promoter fused to *lacZ* (*attP* site, FRT, Tc^R)	This study
pCTXter-P*lepB-lacZ*	miniCTX-T*rrnB-lacZ* harboring the *lepB* promoter fused to *lacZ* (*attP* site, FRT, Tc^R)	This study
pKT25	Cloning and expression vector, encodes the T25 fragment (amino acids 1–224 of CyaA) (Km^R)	(Karimova *et al.*, 1998)

pKT25-HptA	Fusion of *hptA* to *cya* gene T25 fragment in pKT25 (Km^R)	This study
pKT25-HptB	Fusion of *hptB* to *cya* gene T25 fragment in pKT25 (Km^R)	This study
pUT18c	Cloning and expression vector, encodes the T18 fragment (amino acids 225–399 of CyaA) (Ap^R)	(Karimova *et al.*, 1998)
pUT18c-2583-D1	Fusion of sequence encoding D1 domain (834-992 aa) of PA2583 to *cya* gene T18 fragment in pUT18c (Ap^R)	This study
pUT18c-SagS-D1	Fusion of sequence encoding D1 domain (663-786 aa) of SagS to *cya* gene T18 fragment in pUT18c (Ap^R)	This study
pUT18c-RocA1-D2	Fusion of sequence encoding D2 domain of RocA1 to *cya* gene T18 fragment in pUT18c (Ap^R)	(Kulasekara *et al.*, 2004)
pUT18c-RocA2-D2	Fusion of sequence encoding D2 domain of RocA2 to *cya* gene T18 fragment in pUT18c (Ap^R)	(Sivaneson *et al.*, 2011)
pUT18c-RocA3-D2	Fusion of sequence encoding D2 domain (1-134 aa) of RocA3 to *cya* gene T18 fragment in pUT18c (Ap^R)	This study
pUT18c-RocR-D2	Fusion of sequence encoding D2 domain of RocR to *cya* gene T18 fragment in pUT18c (Ap^R)	(Kulasekara *et al.*, 2004)
pUT18c-RocR3-D2	Fusion of sequence encoding D2 domain (1-136 aa) of RocR3 to *cya* gene T18 fragment in pUT18c (Ap^R)	This study

Supplementary references

Baynham PJ, Ramsey DM, Gvozdyev BV, Cordonnier EM, Wozniak DJ. (2006) The *Pseudomonas aeruginosa* ribbon-helix-helix DNA-binding protein AlgZ (AmrZ) controls twitching motility and biogenesis of type IV pili. *J Bacteriol* **188**: 132–140.

Elsen, S., Simon, V., and Attrée, I. (2024) Cross-regulation and cross-talk of conserved and accessory two-component regulatory systems orchestrate *Pseudomonas* copper resistance. *PLoS Genet* **20**: e1011325.

Hoang, T.T., Karkhoff-Schweizer, R.R., Kutchma, A.J., and Schweizer, H.P. (1998) A broad-host-range Flp-FRT recombination system for site-specific excision of chromosomally-located DNA sequences: application for isolation of unmarked Pseudomonas aeruginosa mutants. *Gene* **212**: 77–86.

Karimova, G., Pidoux, J., Ullmann, A., and Ladant, D. (1998) A bacterial two-hybrid system based on a reconstituted signal transduction pathway. *Proc Natl Acad Sci USA* **95**: 5752–5756.

Kessler, B., Lorenzo, V. de, and Timmis, K.N. (1992) A general system to integrate *lacZ* fusions into the chromosomes of gram-negative eubacteria: regulation of the Pm promoter of the TOL plasmid studied with all controlling elements in monocopy. *Molec Gen Genet* **233**: 293–301.

Kos, V.N., Déraspe, M., McLaughlin, R.E., Whiteaker, J.D., Roy, P.H., Alm, R.A., et al. (2015) The Resistome of *Pseudomonas aeruginosa* in Relationship to Phenotypic Susceptibility. *Antimicrob Agents Chemother* **59**: 427–436.

Kovach, M.E., Elzer, P.H., Steven Hill, D., Robertson, G.T., Farris, M.A., Roop, R.M., and Peterson, K.M. (1995) Four new derivatives of the broad-host-range cloning vector pBBR1MCS, carrying different antibiotic-resistance cassettes. *Gene* **166**: 175–176.

Kulasekara, H.D., Ventre, I., Kulasekara, B.R., Lazdunski, A., Filloux, A., and Lory, S. (2004) A novel two-component system controls the expression of Pseudomonas aeruginosa fimbrial *cup* genes: Regulation of P. aeruginosa Cup fimbriae by Roc1. *Mol Microbiology* **55**: 368–380.

Newman, J.R., and Fuqua, C. (1999) Broad-host-range expression vectors that carry the l-arabinose-inducible *Escherichia coli araBAD* promoter and the *araC* regulator. *Gene* **227**: 197–203.

Rietsch, A., Vallet-Gely, I., Dove, S.L., and Mekalanos, J.J. (2005) ExsE, a secreted regulator of type III secretion genes in *Pseudomonas aeruginosa*. *Proc Natl Acad Sci USA* **102**: 8006–8011.

Sivaneson, M., Mikkelsen, H., Ventre, I., Bordi, C., and Filloux, A. (2011) Two-component regulatory systems in Pseudomonas aeruginosa: an intricate network mediating fimbrial and efflux pump gene expression. *Mol Microbiol* **79**: 1353–66.

Table S2. Primers used in this work

Name	Sequence (5' => 3')	Use
**pEXG2-mut-PA4080-sF1**	GGTCGACTCTAGAGGATCCCCTCT GAGGGATATGCAAGTTTCCT	*rocA3* deletion
**pEXG2-mut-PA4080-sR1**	GCGATGTAGGGACATCCTTGC	*rocA3* deletion
**pEXG2-mut-PA4080-sF2**	GCAAGGATGTCCCTACATCGCTGC CGGATGCCCTGACGGAT	*rocA3* deletion
**pEXG2-mut-PA4080-sR2**	ACCGAATTCGAGCTCGAGCCCGAT GTGGCGGAAATCGACGCC	*rocA3* deletion
**pEXG2-mut-rocA1-sF1**	GGTCGACTCTAGAGGATCCCCGAT GTCGACGTGTCCGCAGG	*rocA1* deletion
**pEXG2-mut-rocA1-sR1**	CAGGACGGTATGCATAAATTCGA	*rocA1* deletion
**pEXG2-mut-rocA1-sF2**	GAATTTATGCATACCGTCCTGTCGC TGATCCACTGACCTGGC	*rocA1* deletion
**pEXG2-mut-rocA1-sR2**	ACCGAATTCGAGCTCGAGCCCGAA TTTCCGGCCGACCGAGG	*rocA1* deletion
**pEXG2-mut-rocA2-sF1**	GGTCGACTCTAGAGGATCCCCGAA GCTCTAAGCGATAGCGCC	*rocA2* deletion
**pEXG2-mut-rocA2-sR1**	TATCAGGATACGGCTCATTGTCT	*rocA2* deletion
**pEXG2-mut-rocA2-sF2**	ACAATGAGCCGTATCCTGATAGAG TTCGCCAAACGCAATACGC	*rocA2* deletion
**pEXG2-mut-rocA2-sR2**	ACCGAATTCGAGCTCGAGCCCGTT GCAGGCCAGCAGGCGAC	*rocA2* deletion
**pEXG2-mut-PA4080-sF1**	GGTCGACTCTAGAGGATCCCCTCT GAGGGATATGCAAGTTTCCT	*rocA3-D58A* mutation
**pEXG2-PA4080-D58A-sR1**	GGCATCGTATAGGCGACGATAAC	*rocA3-D58A* mutation
**pEXG2-PA4080-D58A-sF2**	GTTATCGTCGCCTATACGATGCC	*rocA3-D58A* mutation

**pEXG2-mut-PA4080-sR2**	ACCGAATTCGAGCTCGAGCCCGAT GTGGCGGAAATCGACGCC	*rocA3-D58A* mutation
**pEXG2-PA2583-Sp-sF1**	GTCGACTCTAGAGGATCCCCTAGAT CTATAGAGTTCCAATGAAGT	P*BAD* insertion
**pEXG2-PA2583-Sp-sR1**	ACTAGCGCTCTGGCAGCCGCCTTAT	P*BAD* insertion
**pEXG2- PA2583-Sp-sF2**	GGCGGCTGCCAGAGCGACTAGTAG TGTCGACAGGCGTGGGTTC	P*BAD* insertion
**pEXG2-PA2583-Sp-sR2**	CCGAATTCGAGCTCGAGCCCTGCG AATCGAACGGGGCAC	P*BAD* insertion
**pCTXter-cupB1-lacZ-sF**	GATATCGAATTCCTGCAGCCCTCCA TCCGGAATGCGAGTGGG	P*cupB1-lacZ* fusion
**pCTXter-cupB1-lacZ-sR**	GCTAGTTAGTTAGGATCCCCCCATC TGATTTCCTTTGGAGTTGTG	P*cupB1-lacZ* fusion
**pCTXter-cupC1-lacZ-sF**	GATATCGAATTCCTGCAGCCCAGG CAAACTAAGTGCGTTCGAAA	P*cupC1-lacZ* fusion
**pCTXter-cupC1-lacZ-sR**	GCTAGTTAGTTAGGATCCCCCCATG ATTGAGCTTCCTTTTGACAG	P*cupC1-lacZ* fusion
**pCTXter-PA4080-lacZ-sF**	GATATCGAATTCCTGCAGCCCGCTC GTCCTGGGTCTCGTG	P*rocA3-lacZ* fusion
**pCTXter-PA4080-lacZ-sR**	GCTAGTTAGTTAGGATCCCCCATCC TTGCGTCTCCTTAAGC	P*rocA3-lacZ* fusion
**pCTXter-mexA-lacZ-sF**	GATATCGAATTCCTGCAGCCCAGCT CGCGGATCTTCCG	P*mexA-lacZ* fusion
**pCTXter-mexA-lacZ-sR**	GCTAGTTAGTTAGGATCCCCCATA GCGTTGTCCTCATGAGC	P*mexA-lacZ* fusion
**pCTXter-lepB-lacZ-sF**	GATATCGAATTCCTGCAGCCCTCTA GCGCTGCGTCCTGCC	P*lepB-lacZ* fusion
**pCTXter-lepB-lacZ-sR**	GCTAGTTAGTTAGGATCCCCCATG AGGCCCGTACGGAGC	P*lepB-lacZ* fusion
**pJN105-PA4080-sF**	CTAGCGAATTCCTGCAGCCCTTTCC TGGTAACGCTTTCGCCT	*rocA3* expression
**pJN105-PA4080-sR**	CTAGAACTAGTGGATCCCCCTCAG GGCATCCGGCAATAGCC	*rocA3* expression
**pJN105-rocR-sF**	CTAGCGAATTCCTGCAGCCC AACACAGCAGTATATCGCCGGT	*rocR* expression
**pJN105-rocR-sR**	CTAGAACTAGTGGATCCCCC TAGAGGCAGCCGAGGCCAG	*rocR* expression
**pJN105-rocR3-sF**	CTAGCGAATTCCTGCAGCCC ACAATCGCTCCACCATAAAGATTA	IHMA87 *rocR3* expression
**pJN105-rocR3-sR**	CTAGAACTAGTGGATCCCCC GTTGATATCGCAACGACCGCC	IHMA87 *rocR3* expression
**pJN105-hptA-sF**	CTAGCGAATTCCTGCAGCCC GACAGCGCAACGCGCAGTGA	*hptA* expression
**pJN105-hptA-sR**	CTAGAACTAGTGGATCCCCC GAAACCTCTATCTGGTCCGATCT	*hptA* expression
**pKT25-hptA-sF**	CTGCAGGGTCGACTCTAGAG ATGAAAGAGCTTGGTTCGGAATC	pKT25 cloning for BTH

**pKT25-hptB-sR**	TACTTAGGTACCCGGGGATC TCAGCGATAGCGCTGACGTTC	pKT25 cloning for BTH
**pKT25-hptB-sF**	CTGCAGGGTCGACTCTAGAG ATGTCCGCGCCGCATCTCGA	pKT25 cloning for BTH
**pUT18c-PA2583 D1-sR**	GAGCTCGGTACCCGGGGATC TCATTGCGAATCGACGCTCGT	pUT18c cloning for BTH
**pUT18c-PA2583 D1-sF**	ACTGCAGGTCGACTCTAGAG ACCGAGCCTCCGCCCAGC	pUT18c cloning for BTH
**pUT18c-sagSD1-sF**	ACTGCAGGTCGACTCTAGAG ACCCGGGTCCTGCTGGTGGA	pUT18c cloning for BTH
**pUT18c-sagSD1-sR**	GAGCTCGGTACCCGGGGATC CTAGTCGCTCGCGGTGAGCG	pUT18c cloning for BTH
**pUT18c-rocA2D2-sF**	ACTGCAGGTCGACTCTAGAG ATGAGCCGTATCCTGATAGTCG	pUT18c cloning for BTH
**pUT18c-rocA2D2-sR**	GAGCTCGGTACCCGGGGATC CTACGAGCTGGGAAAGTAACTGTA T	pUT18c cloning for BTH
**pUT18c-PA4080D2-sF**	ACTGCAGGTCGACTCTAGAG ATGTCCCTACATCGCATTCGC	pUT18c cloning for BTH
**pUT18c-PA4080D2-sR**	GAGCTCGGTACCCGGGGATC TTACGGCGAGAAATACTGGTTTCG	pUT18c cloning for BTH
**pUT18c-rocR3D2-sF**	ACTGCAGGTCGACTCTAGAG ATGCGGCAGATCAGCGTCCT	pUT18c cloning for BTH
**pUT18c-rocR3D2-sR**	GAGCTCGGTACCCGGGGATC CTAAAGAGCGGGCGGAAGCAGC	pUT18c cloning for BTH
**pBBR-rocS4-sR1**	GACCGGTATAGTCGCTGCTGA	*rocS4* expression
**pBBR-rocS4-sF1**	GATATCGAATTCCTGCAGCCCCTGC CAGAGCGAGTGTCGAC	*rocS4* expression
**pBBR-rocS4-sF2**	TCAGCAGCGACTATACCGGTCGCT ATCTGTCCGCCCAGCATT	*rocS4* expression
**pBBR-rocS4-sR2**	TCTAGAACTAGTGGATCCCCCACG GCAACCAGTCGGGCACT	*rocS4* expression
**pBBR-rocS4c-sR1**	CATGTTAGCAGGTAGATGGATAC	expression of the cytoplasmic portion of RocS4
**pBBR-rocS4c-sF2**	GTATCCATCTACCTGCTAACATGCC CGCCGAACGTATCAAAGC	expression of the cytoplasmic portion of RocS4
**pMMB67HE42 rocS4-F**	AAGAAGGAATATCACAAGTTTGTA CAAAAAAGCAGGCTCCGAAGGAG ATACCATGGCGGCATCGTTGCCGA TG	*rocS4* expression
**pMMB67HE42 rocS4-R**	TCCGCCAAAACAGCCAAGCTGAAT TCACGGCAACCAGTCGGGCACTT	*rocS4* expression
